# Molecular analysis of the reactions in *Salicornia europaea* to varying NaCl concentrations at various stages of development to better exploit its potential as a new crop plant

**DOI:** 10.3389/fpls.2024.1454541

**Published:** 2024-09-03

**Authors:** Andre Fussy, Jutta Papenbrock

**Affiliations:** Institute of Botany, Leibniz University Hannover, Hannover, Germany

**Keywords:** *Salicornia europaea*, gene expression, salt stress, nutritional deficiency, biomarker, osmoregulation, development

## Abstract

Freshwater scarcity demands exploration of alternative resources like saline water and soils. Understanding the molecular mechanisms behind NaCl regulation in potential crop plants becomes increasingly important for promoting saline agriculture. This study investigated the euhalophyte *Salicornia europaea*, analyzing its gene expression, yield, and total phenolic compounds under hydroponic cultivation. We employed five salinity levels (0, 7.5, 15, 22.5, and 30 g/L NaCl) across five harvests at 15-day intervals, capturing plant development. Notably, this design deviated from conventional gene expression studies by recording organ-specific responses (shoots and roots) in plants adapted to long-term salinity treatments at various developmental stages. The highest fresh mass of *S. europaea* was observed four months after germination in 15 g/L NaCl. Identifying a reliable set of reference genes for normalizing gene expression data was crucial due to comparisons across shoots, roots, developmental stages, and salinity levels. A set of housekeeping genes – ubiquitin c (*SeUBC*), actin (*SeActin*) and dnaJ-like protein (*SeDNAJ*) – was identified for this purpose. Interestingly, plants grown without NaCl (0 g/L) displayed upregulation of certain genes associated with a NaCl deficiency related nutritional deprivation. These genes encode a tonoplast Na^+^/H^+^-antiporter (*SeNHX1)*, a vacuolar H^+^-ATPase (*SeVHA-A*), two H^+^-PPases (*SeVP1*, *SeVP2*), a hkt1-like transporter (*SeHKT*), a vinorine synthase (*SeVinS*), a peroxidase (*SePerox*), and a plasma membrane Na^+^/H^+^-antiporter (*SeSOS1*). Other genes encoding an amino acid permease (*SeAAP*) and a proline transporter (*SeProT*) demonstrated marginal or dispersing salinity influence, suggesting their nuanced regulation during plants development. Notably, osmoregulatory genes (*SeOsmP*, *SeProT*) were upregulated in mature plants, highlighting their role in salinity adaptation. This study reveals distinct regulatory mechanisms in *S. europaea* for coping with varying salinity levels. Identifying and understanding physiological reactions and sodium responsive key genes further elucidate the relationship between sodium tolerance and the obligate sodium requirement as a nutrient in euhalophytes.

## Introduction

The Borlaug hypothesis suggests that improved cropping technologies can meet the growing world’s food needs without converting natural landscapes into cropland (Borlaug, 2007). However, evidence from some studies indicates a need for a significant increase in agricultural land to meet future food demands (Ray et al., 2013; Molotoks et al., 2018). Moreover, salinization poses a threat to agricultural yields (Zörb et al., 2019). Investigating salt-tolerant crops for food production and enhancing the salt tolerance of crops, potentially through biotechnological optimization, is therefore an important step toward securing the global food supply. *Salicornia* is consumed in various forms, such as raw, cooked, or marinated. Beyond its culinary uses, it also serves as feed for numerous livestock. Its potential extends to biofuel production, and its constituents have potential applications in the pharmaceutical and cosmetic industries (Ksouri et al., 2012). Regarding cultivation, previous research highlights the advantages and disadvantages of hydroponic systems in terms of their usability for food production (Fussy and Papenbrock, 2022). Here, plant production can occur efficiently in a controlled environment in previously unusable areas close to the consumer under optimized water-saving conditions, assuming that the question of the provision of energy for light and heat requirements is addressed sustainably.

The genus *Salicornia*, commonly known as glasswort or samphire, is composed of succulent plants with articulated stems that are well adapted to saline environments, such as salt marshes (Kadereit et al., 2007). *Salicornia europaea* can tolerate more than 1000 mM (equivalent to 58.44 g/L) NaCl with an optimal salt requirement between 200-400 mM (11.69 - 23.38 g/L) NaCl (Lv et al., 2012a). This presumably corresponds to salt concentrations of areas close to the coastline like mudflats with an average salt content of the oceans of 35 g/L (about 600 mM), taking into account the diluting input of rain. Its leaves are opposite and connate and are usually reduced to small tubercles or scales. The plants feature flowers that are either bisexual or unisexual and are arranged in spicate inflorescences (Scott, 1977). *Salicornia* species exemplify obligate halophytes (euhalophytes). Their not well-defined requirement for an external sodium supply underscores the need for further investigating this group of plants (Grigore, 2021). Its widespread distribution in salt marshes, coastal areas, and salt flats across continents underscores its potential for saline agriculture (Ladeiro, 2012). Due to the greatly reduced morphology of *Salicornia*, the annual halophyte is to be assessed in its state of development primarily on the basis of its size and the appearance of flowers. Therefore, the determination of the changes in fresh mass over fixed periods of time can provide information on the state of development. It was known from previous research that *S. europaea* barely gained any fresh mass after 10 weeks of hydroponic cultivation, had predominantly reached the reproductive phase and subsequently formed greater amounts of lignin up to the 15th week and took on reddish to yellowish colors as a sign of senescence (Turcios et al., 2023). Furthermore, the ambiguous morphological variations and genetic analyses have led to challenges in taxonomic classification, resulting in the genus *Salicornia* being described as a “taxonomic nightmare” (Kadereit et al., 2007).

Plants need to adapt to diverse and fluctuating environmental factors, such as temperature, light conditions, water supply, and nutrient availability. Stress responses are triggered when one or more of these factors diverge from the optimal conditions of plants (Zhu, 2002). The plant kingdom has evolved various stress responses and coping strategies. Analyzing the expression levels of genes that regulate sodium and protect against stress can enhance our understanding of regulatory mechanisms, including adaptation strategies to various salinity levels. Moreover, certain metabolite concentrations have been demonstrated to increase under stress caused by elevated salt levels (Boestfleisch et al., 2014). In a previous study, a pronounced antioxidant activity of the leaves of *Salicornia brachiata* was found, which could be due to the presence of polyphenols, which in turn, as reductones, convert free radicals into more stable products (Daffodil et al., 2013). Confirmatory findings included the identification of polyphenols as important compatible solutes in *S. brachiata* for maintaining osmotic balance, protecting cellular macromolecules, detoxifying cells and scavenging free radicals under drought stress, with their concentration increasing with increasing stress treatments (Parida and Jha, 2013). The determination of the salt concentrations at which the plant is subjected to mild stress, which can simultaneously increase valuable metabolite levels but does not severely impair plant growth itself, and the impact of the time over which the plant is subjected to mild stress is sought in the gene expression reactions of genes related to stress tolerance.

A significant obstacle in studying non-model plants such as *S. europaea* is the limited availability and quality of genomic and transcriptomic sequence data. In this context, gene expression analysis can be used as a pivotal tool for identifying specific protein-encoding genes that fulfill unique functions within an organism, especially in the context of stress management (Jamil et al., 2011). Previous research has demonstrated the utility of understanding stress tolerance genes. For instance, the expression of *SbGSTU* (a tau class glutathione S-transferase) from *Salicornia brachiata* in *Nicotiana benthamiana* has been shown to enhance plant salt stress tolerance in transformed plants (Jha et al., 2011). Similarly, enhanced salt stress tolerance in *N. benthamiana* caused by the expression of *SbSOS1* (salt overly sensitive 1 from *S. brachiata*), which encodes a plasma membrane Na^+^/H^+^ antiporter, has been demonstrated (Jha et al., 2011; Yadav et al., 2012). The *SbNHX1* gene from *S. brachiata* encodes a tonoplast Na^+^/H^+^ antiporter that is increasingly transcribed in response to elevated salinity, and it was possible to identify the associated promoter that contains stress-responsive cis-regulatory motifs (Tiwari et al., 2019). Previously, genes encoding an osmotin protein (*SeOsmP*), a vinorine synthase involved in alkaloid biosynthesis (*SeVinS*), an amino acid permease (*SeAAP*) and a proline transporter (*SeProT*) were shown to be upregulated under salt-free conditions in *S. europaea* (Ma et al., 2013). The researchers argued, in agreement with previous literature, that these observations indicate that a salt-free environment is detrimental to halophytes and that these plants synthesize, transport and accumulate low molecular weight organic compounds such as osmotin, proline, other amino acids, simple sugars, disaccharides, alkaloids and stress proteins in the cytosol and organelles to increase cellular turgor pressure and cell expansion in shoots under salt-free conditions (Ueda et al., 2004; Sokhansanj et al., 2006). These and other genes might therefore serve as salinity-responsive biomarkers for improving yield and tolerance traits as well as for the selection of salt-tolerant genotypes in modern plant breeding, which has previously been shown, e.g., in cotton and olives (Sadder et al., 2021; Han et al., 2023).

It is hypothesized that the roots and shoots of *S. europaea* play distinct roles in salt management, as indicated by their differing gene expression patterns. Furthermore, selected genes are hypothesized to be differentially expressed during the development of roots and shoots, suggesting that they have various functions in these tissues. Among the known salinity-responsive genes in *S. europaea*, those encoding a tonoplast Na^+^/H^+^ antiporter (*SeNHX1)*, a vacuolar H^+^-ATPase (*SeVHA-A*) and two H^+^-PPases (*SeVP1* and *SeVP2*) were selected for analysis (Lv et al., 2012a). A *hkt1-like* gene encoding a sodium transporter involved in Na^+^ homeostasis (*SeHKT*) and a gene encoding a peroxidase (*SePerox*), as well as the aforementioned genes *SeOsmP*, *SeProT*, *SeAAP*, *SeVinS*, and *SeSOS1*, were further selected for analyses based on their responsiveness to varying salt concentrations (Ma et al., 2013). The experimental approach described in this study is unique because it assesses plant responses across a broad range of salinity levels under conditions to which plants have already adapted. This approach increases the knowledge of gene regulatory mechanisms as balanced responses to environmental conditions, comparing them throughout nearly the entire developmental spectrum, from young plantlets to maturity. This approach enhances the understanding of how obligate halophytes such as *S. europaea* maintain growth under long-term varying salt conditions.

## Materials and methods

### Seed germination

*S. europaea* seeds were obtained from a breeder (Joost Bogemans, Scrops Serra Maris bvba, Ninove, Belgium). The agronomic procedures, from sowing to transplanting in hydroponic systems, followed recent methods (Buhmann and Papenbrock, 2013) involving six weeks of preculture in soil after sowing. On 7 July 2022, *S. europaea* var. aprica was transplanted into hydroponic systems at the Institute of Botany, Leibniz University Hannover (LUH), Germany (52°23′42″ N; 9°42′13″ E). The day length was prolonged to 16 h by applying artificial light in the morning (6 to 8 a.m.) and at night (6 to 10 p.m.) (sodium vapor lamps, SON-T Agro 400) to reduce the probability of early flowering induction in *S. europaea* (Ventura et al., 2011).

### Hydroponic cultivation

The experimental setup mirrored the conditions outlined in the seed germination section. Polypropylene containers (400 × 300 × 175 mm, length by width by height; Eurobox, Ant^®^, 131846, Jungheinrich AG, Hamburg, Germany) with a capacity of 14 L were used for hydroponic cultivation. Each container was filled with 13.5 L of modified Hoagland medium adjusted to a pH value between 6 and 6.2, containing 606 mg/L KNO_3_, 944 mg/L Ca(NO_3_)_2_·4H_2_O, 230 mg/L NH_4_H_2_PO_4_, 246 mg/L MgSO_4_·7H_2_O, 3.73 mg/L KCl, 1.55 mg/L H_3_BO_3_, 0.34 mg/L MnSO_4_·H_2_O, 0.58 mg/L ZnSO_4_·7H_2_O, 0.12 mg/L CuSO_4_·5H_2_O, 0.12 mg/L MoNa_2_O_4_·2H_2_O, and 9.16 mg/L Fe-EDDHA (0.56 mg Fe/L) (Hoagland and Arnon, 1950). The hydroponic systems utilized a nutrient solution with salt concentrations of 0, 7.5, 15, 22.5, and 30 g/L NaCl. The salinity of the growth media was adjusted using NaCl (sodium chloride ≥ 99.8% purity, with anti-caking agent, Carl Roth GmbH + Co. KG, Karlsruhe, Germany), and salt concentrations were regularly checked with a multimeter (Multi 350i, Xylem Analytics Germany Sales GmbH & Co. KG., Weilheim, Germany) before and after changing the media, ensuring consistency. Daily checks were conducted to maintain the nutrient solution level at 13.5 L by adding water as needed. Light intensity was monitored at various times using a quantum meter (Model MQ-200, Apogee Instruments Inc., Logan, Utah, USA), ranging between 198 and 637 µmol m^-2^ s^-1^, influenced by weather conditions and supplemental artificial lighting. The nutrient medium was refreshed every 15 d following each harvest. To secure the young plants within the setup, they were anchored in the lids of the containers using foam, with a distribution of five plants per box.

### Sampling and weight-related measurements

After five weeks of hydroponic cultivation, the initial harvest of plants from each treatment group took place on 11 August 2022. Subsequent samples were collected at 15 d intervals. At each harvest, the fresh mass of *S. europaea* shoots was recorded. The fresh weight of the roots could not be recorded, as the roots partly grew together at later stages of development in the deep water-culture, and complete separation would not have been possible without damaging the plants remaining in culture. For the purpose of gene expression and metabolite analyses, segments of both shoots and roots from each harvested plant were immediately frozen separately in liquid nitrogen. The tissue samples were selected from various positions to ensure comprehensive analysis.

### Determination of total phenolic compounds

The preparation of the samples and extraction of phenolic compounds followed a previously described method (Boestfleisch et al., 2014). The concentration of total phenolic compounds in the extract was determined using Folin-Ciocalteu reagent. The exact procedure for quantifying total phenolic compounds was previously described (Dudonné et al., 2009). The absorbance at 765 nm was measured using a microplate reader (Synergy Mx, BioTek Germany, Bad Friedrichshall, Germany) equipped with 96-well microplates (Sarstedt AG & Co., Nümbrecht, Germany). The results are expressed as gallic acid equivalents (µg GAE/mg FW).

### Gene expression analysis

#### RNA isolation

Total RNA was isolated from the leaves and roots of plants that had been finely pulverized in liquid nitrogen. Approximately 100 mg of plant powder was processed using the Plant RNA Isolation Kit (MACHEREY-NAGEL GmbH & Co. KG, Düren, Germany) according to the manufacturer’s instructions. The concentration of the extracted RNA was determined photometrically, and its quality was assessed by examining the ratio of photometric absorbance at 260 nm to that at 230 nm, as well as through gel electrophoresis ((Heptinstall and Rapley, 2000).

#### cDNA synthesis of the first strand

The synthesis of the first strand of cDNA was adapted based on a previously described method and performed in a PCR cycler (peqSTAR XS, VWR International GmbH, Darmstadt, Germany), with certain steps executed in a water bath at 70°C (Krug and Berger, 1987). Reactions were performed with RNA samples, a combination of random nonamer and oligo (dT) primers, dNTPs (dNTP-Set, Life Technologies GmbH, Darmstadt, Germany), RT buffer, and M-MLV reverse transcriptase (Promega GmbH, Walldorf, Germany). The resulting cDNA was preserved at -20°C for long-term storage or at 4°C for short-term use.

#### Gene expression analysis based on synthesized cDNA

Quantification of transcribed RNA was conducted using quantitative real-time PCR (RT-qPCR) with the intercalating dye SYBR Green, included in the qPCR Mastermix (Invitrogen™ Platinum™ SYBR™ Green qPCR SuperMix-UDG, Life Technologies GmbH). The qPCR Mastermix, along with primers and template, contained all necessary components for the qPCR. Primers were designed based on gene sequences and functional annotations from prior studies on *S. europaea* (Lv et al., 2012a; Ma et al., 2013; Xiao et al., 2015). The PCR protocol consisted of an initial step of 2 min at 50°C, followed by 2 min at 95°C, and then 40 cycles of denaturation at 95°C for 15 sec and annealing/extension at 60°C for 1 min. Specificity of the amplification was confirmed by melting curve analysis from 60°C to 95°C. Gene-specific cDNA quantities were determined from threshold cycle (Ct) values, normalized against a reference gene cDNA as an internal standard. Expression levels were normalized using ΔCt=Ct−Ctr and quantified as 2^-ΔCt^. Further normalization with the control values ΔΔCt=Ct–Ctc provided a relative quantification: 2^-ΔΔCt^ (Livak and Schmittgen, 2001). qPCR was performed on an ABI 7300 (Real-Time PCR System 7300, Applied Biosystems, Foster City, CA 94404, USA).

#### Selection of genes and primers for reference genes and genes of interest

Primer design for RT-qPCR assays was carefully optimized, considering primer length, GC content, melting temperature, and avoidance of dimer formation (Kibbe, 2007; Lu et al., 2017). Primers and genes were selected based on their roles in salt management and expected differential expression across varying salinity levels (Lv et al., 2012a; Ma et al., 2013). Detailed information on primer sets, genes, primer sequences, and cellular functions of reference genes and genes of interest for *S. europaea* is provided in **Supplementary Table 1**. Primers ranged from 19 to 25 base pairs, with a GC content of 40–60% and melting temperatures between 62°C and 66°C. The binding specificity was confirmed against transcriptomic data from referenced studies. Reference gene selection for *S. europaea*, especially under different salinity levels and developmental stages, was guided by previous research (Xiao et al., 2015). Potential candidates included α-tubulin (*Seα-tub*), ubiquitin c (*SeUBC*), actin (*SeActin*), and ubiquitin-conjugating enzyme (*SeUBQ*), as well as elongation factor 1-α (*SeEF1-α*), clathrin adaptor complex (*SeCAC*), tip41-like protein (*SeTIP41*), and dnaJ-like protein (*SeDNAJ*). The suitability of these reference genes was assessed using the geNorm function (Currie, 1991), adapted for use in R-Studio to identify the best combination based on minimal variance between samples (smallest M values).

### Statistical methods

Given the biological context of the study, data variance heterogeneities were expected. To address this, generalized linear models were employed for analyzing data with heterogeneous variances. For each trait investigated, a factorized variable was created, followed by ANOVA to assess overall differences. Pairwise mean comparisons across the two independent variables, “NaCl treatment” and “harvest group,” were conducted using the Tukey *post hoc* test. If ANOVA results for a parameter—or gene expression of a specific gene relative to its organ—showed significant interaction between the two independent variables (harvest group and NaCl concentration), significance levels averaged over harvest groups or NaCl concentrations were not interpreted. This approach was used to account nested effect structures that could affect the explanatory values of individual variables within the analysis of a statistical model, potentially complicating or invalidating biological interpretations (Hoshmand, 2006).

## Results

For an easier representation, a descriptive evaluation summarizes main findings of the analyzed data (**Supplementary Table 2**).

### General appearance and fresh mass of differently developed shoots of *S. europaea* in response to various salt concentrations

**Figure 1:**
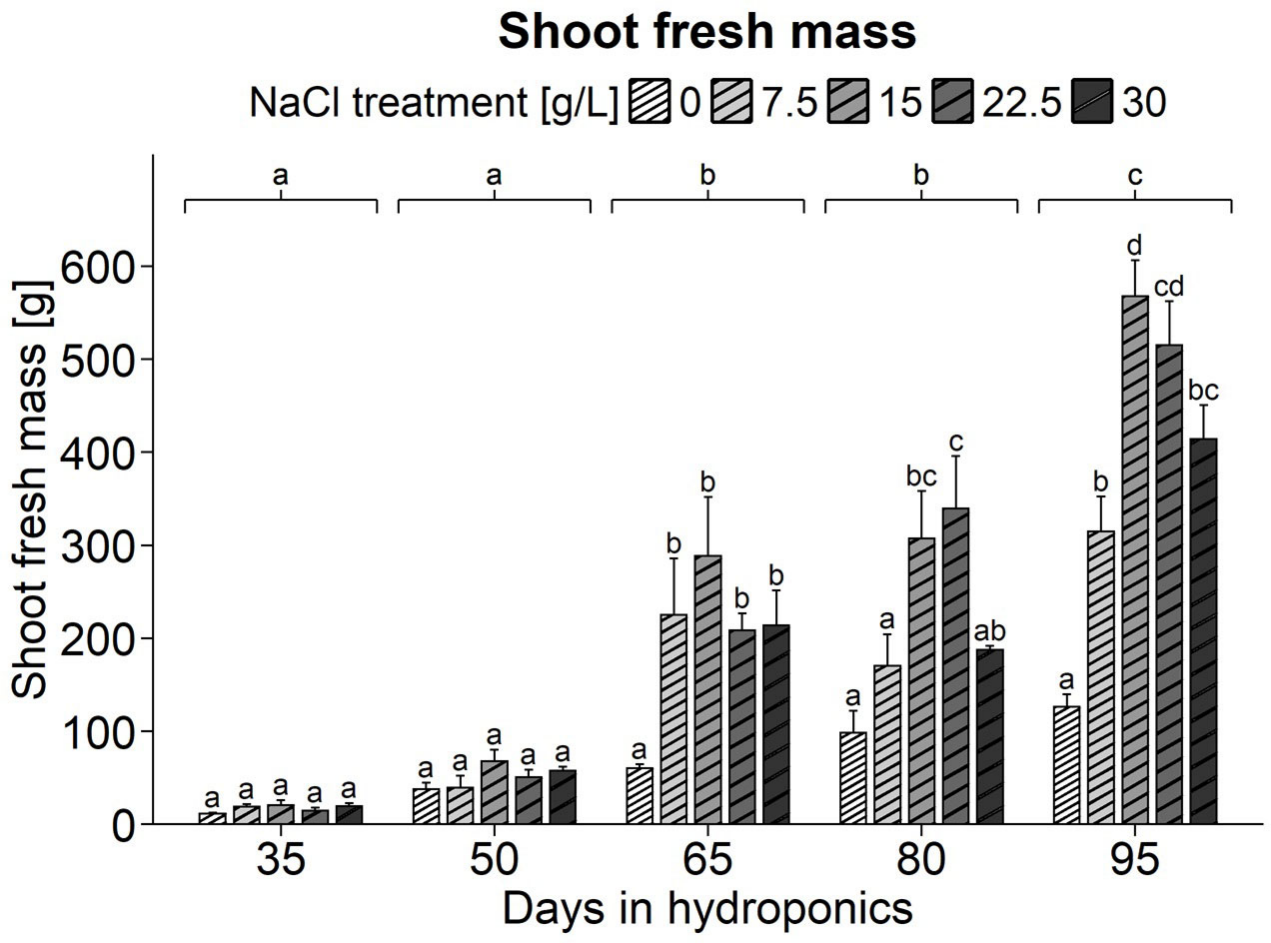
Fresh weight (g) of *S. europaea* shoots from various NaCl treatments (0, 7.5, 15, 22.5, and 30 g/L; equals 0, 128, 257, 385, and 513 mM) and developmental stages (hydroponics for 35, 50, 65, 80 and 90 d). The graphs show the means ± SEs of three replicates (74 plants in total; N.A. for one plant in the 30 g/L NaCl treatment in the fourth harvest group). Similar letters denote no significant difference (P value > 0.05).

Examination of a series of images revealed that plants grown under 0 g/L NaCl remained smaller than those treated with NaCl. Their shoot axes appeared narrower, thinner, and had fewer branches (**Supplementary Figure 1**). Mortaring these plants in liquid nitrogen was more challenging, likely due to higher fiber content indicating greater lignification. Additionally, these plants appeared darker and entered the reproductive phase prematurely, as shown by the emergence of small white flowers. In contrast, *S. europaea* exhibited enhanced growth at 15 g/L NaCl, with visibly thicker stems and denser growth. The assessment of shoot fresh mass in *S. europaea*, focusing on the impact of NaCl treatments across different harvest groups, revealed more pronounced differences as plants developed. One-way ANOVA indicated significant effects of NaCl treatment, harvest group, and their interaction on shoot fresh mass (**Supplementary Table 3**). Detailed observation of **Figure 1** revealed no significant differences in shoot fresh mass due to NaCl treatments in young plants after 35 and 50 d of hydroponic cultivation, although the 0 g/L NaCl-treated plants already had the lowest shoot fresh mass. After 65 d of cultivation, plants treated with 0 g/L NaCl had a shoot fresh mass of 60.33 g, significantly lower than the 208.33 to 288.33 g observed in plants treated with 7.5 to 30 g/L NaCl. This trend continued in subsequent harvests, with plants in the 0 g/L NaCl treatment consistently having the lowest fresh mass, followed by those in the 7.5 g/L NaCl treatment. On average, the highest fresh mass was observed at 15 g/L NaCl, which tended to decrease with higher salt concentrations.

### Determination of total phenolic compounds in differently developed shoots and roots of *S. europaea* in response to various salt concentrations

**Figure 2:**
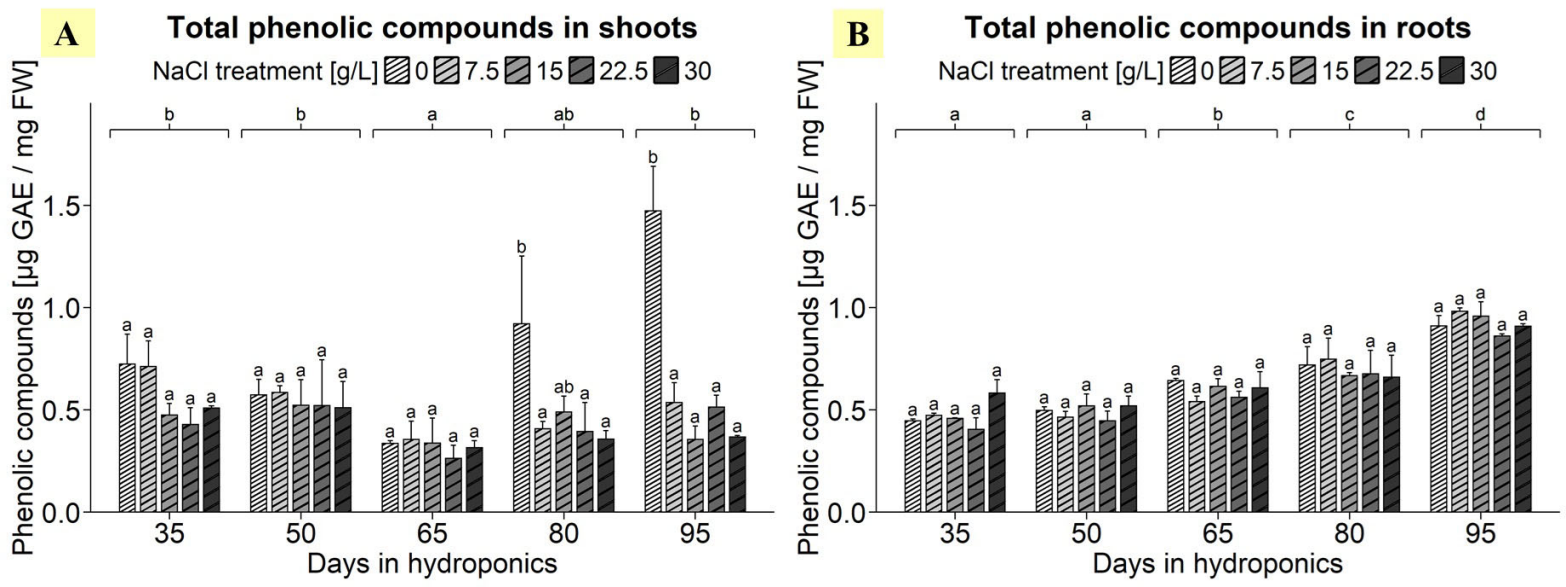
Total phenolic compound contents in **(A)** shoots and **(B)** roots of *S. europaea* from various NaCl treatments (0, 7.5, 15, 22.5, and 30 g/L; equals 0, 128, 257, 385, and 513 mM) and developmental stages (hydroponics for 35, 50, 65, 80 and 90 d). The graphs show the means ± SEs of three replicates (74 plants in total; N.A. for one plant in the 30 g/L (= 513 mM) NaCl treatment in the fourth harvest group). Similar letters denote no significant difference (P value > 0.05).

ANOVA highlighted the significant effects of harvest group, NaCl treatment, and their interaction on the total phenolic compound content in the shoots of *S. europaea* (**Supplementary Table 3**). As shown in **Figure 2A**, shoots treated with 0 g/L NaCl mostly exhibited elevated total phenolic compound contents compared to the higher NaCl concentrations in various harvest groups. Notably, after cultivation for 95 d, the phenolic content of shoots from the 0 g/L NaCl treatment group reached 1.47 µg GAE/mg FW, significantly surpassing the levels observed in shoots harvested from the other NaCl treatment groups, which ranged between 0.36 and 0.54 µg GAE/mg FW. In the 0 g/L NaCl treatment group a decrease in the total phenolic compound content was detected after 65 d, with an average of 0.33 µg GAE/mg FW. Additionally, the analysis revealed a progressive decrease in the total phenolic compound content of shoots with increasing salinity, with the 0 g/L NaCl treatment exhibiting the greatest total phenolic compound content, at 0.81 µg GAE/mg FW, which was 56-98% greater than that in the shoots of the other NaCl treatment groups (**Supplementary Table 4**). In terms of the roots, the ANOVA results indicated that the harvest group had a significant influence on the total phenolic compound content in *S. europaea*, but results were not significantly influenced by the NaCl treatment or their interaction term (**Supplementary Table 3**). **Figure 2B** revealed, that a consistent trend of increasing total phenolic compound content occurred in the roots as the plants developed, with plants cultivated for 95 d showing a peak in the mean total phenolic compound content of approximately 0.91 µg GAE/mg FW, which was significantly greater than that in the roots during earlier development. Similarly, a significant difference was found between roots of plants cultivated for 80 d, which averaged 0.70 µg GAE/mg FW, and those of earlier harvest groups. Moreover, the results showed harvest group-independent NaCl treatment effects, with average phenolic compound contents in roots exhibiting minimal variation across NaCl treatments, ranging between 0.59 and 0.65 µg GAE/mg FW (**Supplementary Table 4**).

### Gene expression analysis - reference gene selection

**Figure 3:**
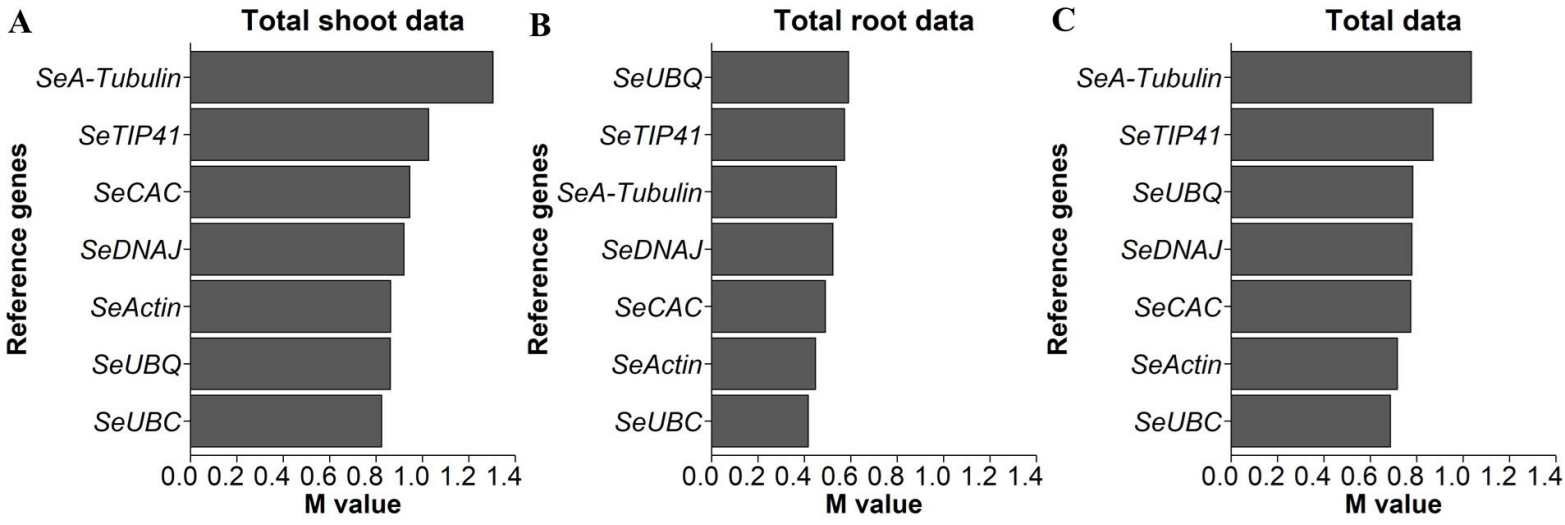
M values based on the geNorm function, with smaller values indicating greater stability and, therefore, underlying genes as better options for normalization of qPCR data for expression levels of indicated candidate genes in *S. europaea* **(A)** shoots, **(B)** roots and **(C)** both organs. The expression levels were normalized to the RNA concentrations, and testing was performed across the different NaCl treatments and harvest groups of the main experiment (74 plants per gene; N.A. for one plant in the 30 g/L (= 513 mM) NaCl treatment in the fourth harvest group).

Regarding the equal usability of the reference genes in the root and shoot organs, it was noticeable that the shoot data had greater variance with regard to the expression levels of the potential reference genes. For the geNorm analysis, *SeEF1-α* was omitted due to poor amplification quality and dimer formation. **Figure 3A** shows that, for shoot gene expression data, *SeUBC*, *SeUBQ*, *SeActin*, and *SeDNAJ* had the lowest M values. According to the root gene expression data shown in **Figure 3B**, *SeUBC*, *SeActin*, *SeCAC*, and *SeDNAJ* had the smallest M values. A combined analysis of both shoot and root data, presented in in **Figure 3C**, confirmed that *SeUBC*, *SeActin*, *SeCAC*, and *SeDNAJ* collectively exhibited the lowest M values, making them optimal for normalization across both tissue types.

To confirm the results, the variances of the expression data of the reference gene candidates for shoot, root and total tissue of *S. europaea* were evaluated again within the individual harvest groups using the geNorm function, as shown separately (**Supplementary Figure 2**). It can be concluded from the detailed analysis that smaller M values for root data were obtained with *SeUBC*, *SeTIP41*, *SeDNAJ*, *SeCAC* and *SeActin* than with *SeA-tubulin* and *SeUBQ*, as *SeTIP41* significantly increased in the data at harvest after 65 d, and *SeCAC* was excluded due to poor PCR performance. According to the shoot data, the analysis reveals that *SeUBC*, *SeActin*, *SeDNAJ*, *SeTIP41* and *SeUBQ* produce lower M values than *SeCAC* and *SeA-tubulin*. However, in the 50 d harvest data, *SeTIP41* performed the worst, *SeCAC* was again excluded due to its lower PCR performance, and *SeUBQ* produced higher M values in the root samples. In the total samples from both shoot and root tissues of *S. europaea*, *SeUBC* showed the lowest M values most frequently among the 5 harvests, followed by *SeTIP41* and *SeActin*; the former was partly unsuitable for root samples, and *SeDNAJ* was in the midfield in terms of M values but was suitable for all individual conditions.

### Gene expression analysis - investigation of genes of interest

#### Gene expression of *SeNHX1*, *SeVP1*, *SeVP2*, *SeVHA-A*, *SeHKT* and *SeSOS1* in differently developed shoots and roots of *S. europaea* in response to various salt concentrations

**Figure 4:**
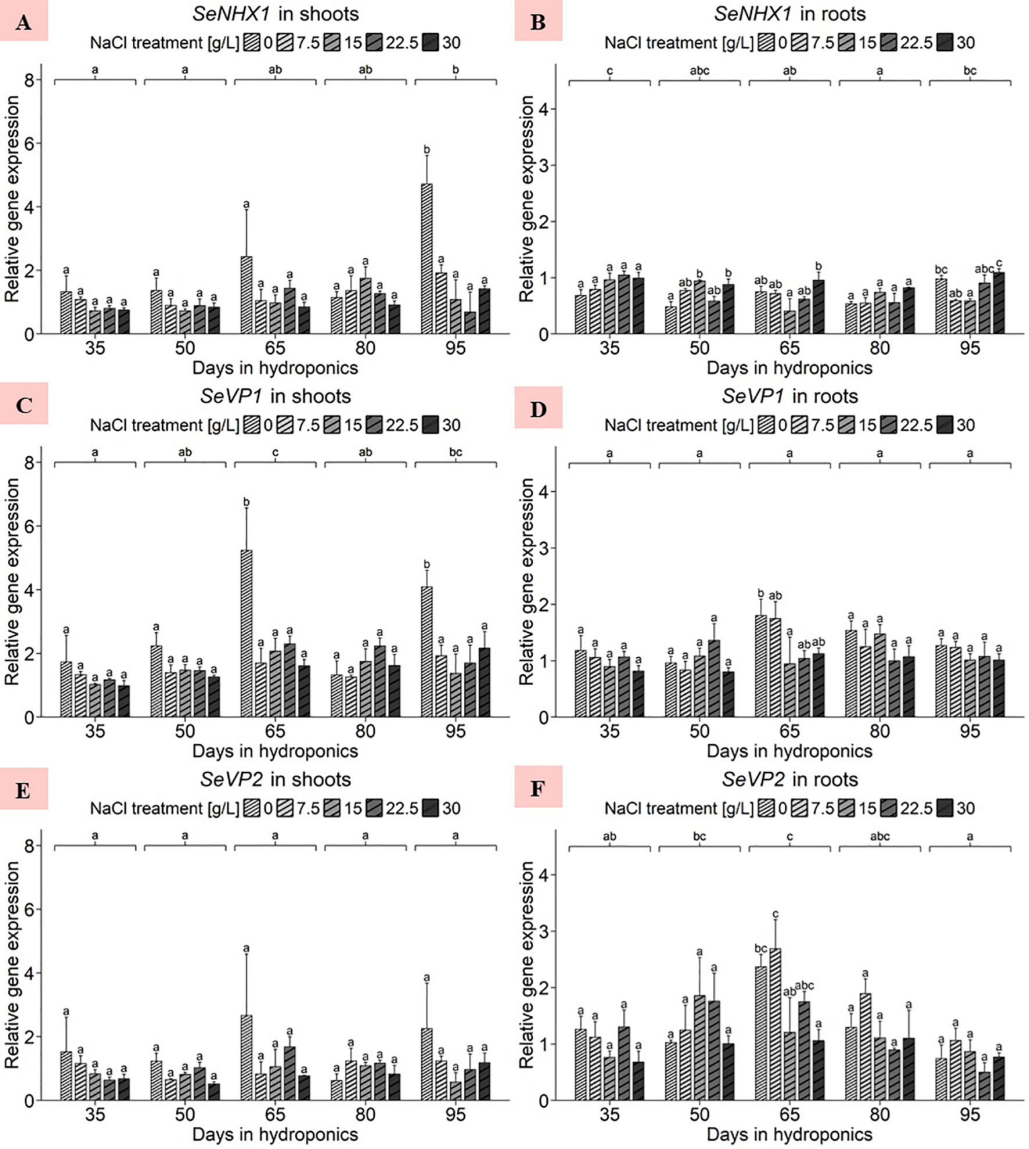
Gene expression analysis of *SeNHX1*, *SeVP1* and *SeVP2* in **(A, C, E)** shoots and **(B, D, F)** roots of *S. europaea* from various NaCl treatments (0, 7.5, 15, 22.5, and 30 g/L; equals 0, 128, 257, 385, and 513 mM) and developmental stages (hydroponics for 35, 50, 65, 80 and 90 d). Expression levels were normalized to the mean *SeUBC*, *SeActin* and *SeDNAJ* reference gene expression levels and RNA concentrations. The graphs show the means ± SEs of three replicates (74 plants in total; N.A. for one plant in the 30 g/L (= 513 mM) NaCl treatment in the fourth harvest group). Similar letters denote no statistical significance (P value > 0.05).

The ANOVA of shoot data revealed significant effects of the harvest group and the NaCl gradient as well as of their interaction term on the gene expression of *SeNHX1* (**Supplementary Table 3**). For the shoots, a significant increase in the gene expression of *SeNHX1* was observed after 95 d of cultivation at 0 g/L NaCl compared to other NaCl treatments of the same harvest, reaching a peak of 4.71 units, as illustrated in **Figure 4A**. Broadening the scope, the harvest after 95 d emerged as the zenith of expression averaged across NaCl treatments, reaching approximately 1.96 relative units. Conversely, plants cultivated for 35 d showed the lowest shoot *SeNHX1* expression, averaging 0.94 relative units. The analysis revealed that shoot expression averaged across all harvests was most pronounced at 0 g/L NaCl, with an average of 2.2 relative units, with the lowest average expression observed at 30 g/L NaCl, with approximately 0.95 relative units (**Supplementary Table 4**). A continuous decrease in *SeNHX1* gene expression was observed in shoots with increasing NaCl concentrations. Moreover, ANOVA of root data revealed significant effects of harvest group, NaCl treatment, and their interaction term on *SeNHX1* expression (**Supplementary Table 3**). As shown in **Figure 4B**, significant NaCl treatment effects in different directions were observed after 50, 65 and 95 d of cultivation. The harvest after 35 d, when averaged across NaCl levels, increased to approximately 0.89 relative units. The variations between harvests were small, with plants cultivated for 80 d exhibiting the lowest average expression, approximating 0.64 relative units. The analysis highlighted the expression response in roots to various salinity levels, with a peak at 30 g/L NaCl of 0.95 relative units, while the lowest expression levels were observed at 0 and 7.5 g/L NaCl, approximately 0.68 relative units (**Supplementary Table 4**). Thus, a constant increase in the gene expression of *SeNHX1* with increasing NaCl concentration was observed in the roots but not in the shoots.

The ANOVA of shoot data revealed significant effects of harvest group, NaCl treatment and their interaction term on *SeVP1* gene expression (**Supplementary Table 3**). With the exception of shoots after 80 d of cultivation, the gene expression of *SeVP1* in plants treated with 0 g/L NaCl was consistently above the expression levels of the other NaCl treatments, as shown in **Figure 4C**. Moreover, when the expression levels were averaged across the NaCl treatments, the harvest after 65 d exhibited an increase in *SeVP1* gene expression of 2.58 relative units. In 0 g/L NaCl treatments averaged across all harvests, an increase in gene expression levels in the shoots was found, averaging 2.92 relative units, which differed from the relatively consistent expression levels observed in higher NaCl concentrations, ranging from 1.52 to 1.77 relative units (**Supplementary Table 4**). Focusing on the root data, the ANOVA findings revealed significant harvest group effects only (**Supplementary Table 3**). As shown in **Figure 4D**, plant gene expression after cultivation for 65 d at 0 g/L NaCl significantly increased to 1.80 relative units. Except for the data after 50 d of cultivation, the plants treated with 0 g/L NaCl had consistently greater gene expression levels than those treated with elevated NaCl concentrations. After averaging the data across the NaCl treatments, cultivation for 65 d resulted in an increase in the expression level to 1.33 relative units, although the difference in the mean expression between the harvests was not significant. Similar to the trends observed in the shoots, the roots of plants grown in 0 g/L NaCl averaged across all harvests exhibited a peak in gene expression, with an average of 1.35 relative units, which was significantly greater than that of plants treated with 30 g/L NaCl (**Supplementary Table 4**). This downward trend in *SeVP1* gene expression was observed almost continuously with increasing NaCl concentration.

Initially, the ANOVA revealed no significant effects of harvest group, NaCl gradient or the interaction term of the two variables on *SeVP2* gene expression in the shoots of *S. europaea* (**Supplementary Table 3**). Within the detailed scope of **Figure 4E**, with the exception of the harvest after 80 d, the gene expression of *SeVP2* was consistently highest in plants treated with 0 g/L NaCl. The analysis revealed that the shoot response to varying salinity averaged across the harvests, with the expression being most pronounced at 0 g/L NaCl, averaging 1.66 relative units, whereas the lowest average expression was observed at 30 g/L NaCl, at 0.92 relative units (**Supplementary Table 4**). In roots, the ANOVA for *SeVP2* revealed significant effects of harvest group and NaCl treatment on gene expression but not of their interaction term (**Supplementary Table 3**). As shown in **Figure 4F**, *SeVP2* gene expression increased the most in the roots of plants treated with 7.5 g/L NaCl for 65 d, reaching approximately 2.69 relative units. A comparison of the cultivation periods averaged across the NaCl treatments revealed that the mean *SeVP2* expression in the roots of the plants cultivated for 65 d was approximately 1.81 relative units, which was significantly greater than that in the roots of the plants cultivated for 95 d. Observing the mean gene expression levels in the roots of *S. europaea* over the experimental period revealed a pattern close to a normal distribution. The analysis showed different reactions of *S. europaea* roots to salinity averaged across harvests, with peaking *SeVP2* expression levels observed in the 7.5 g/L NaCl treatment (with 1.60 relative units) and 0 g/L NaCl treatment (with 1.34 relative units), while significantly lower expression occurred in the roots of plants cultivated in 30 g/L NaCl (with 0.92 relative units) (**Supplementary Table 4**).

**Figure 5:**
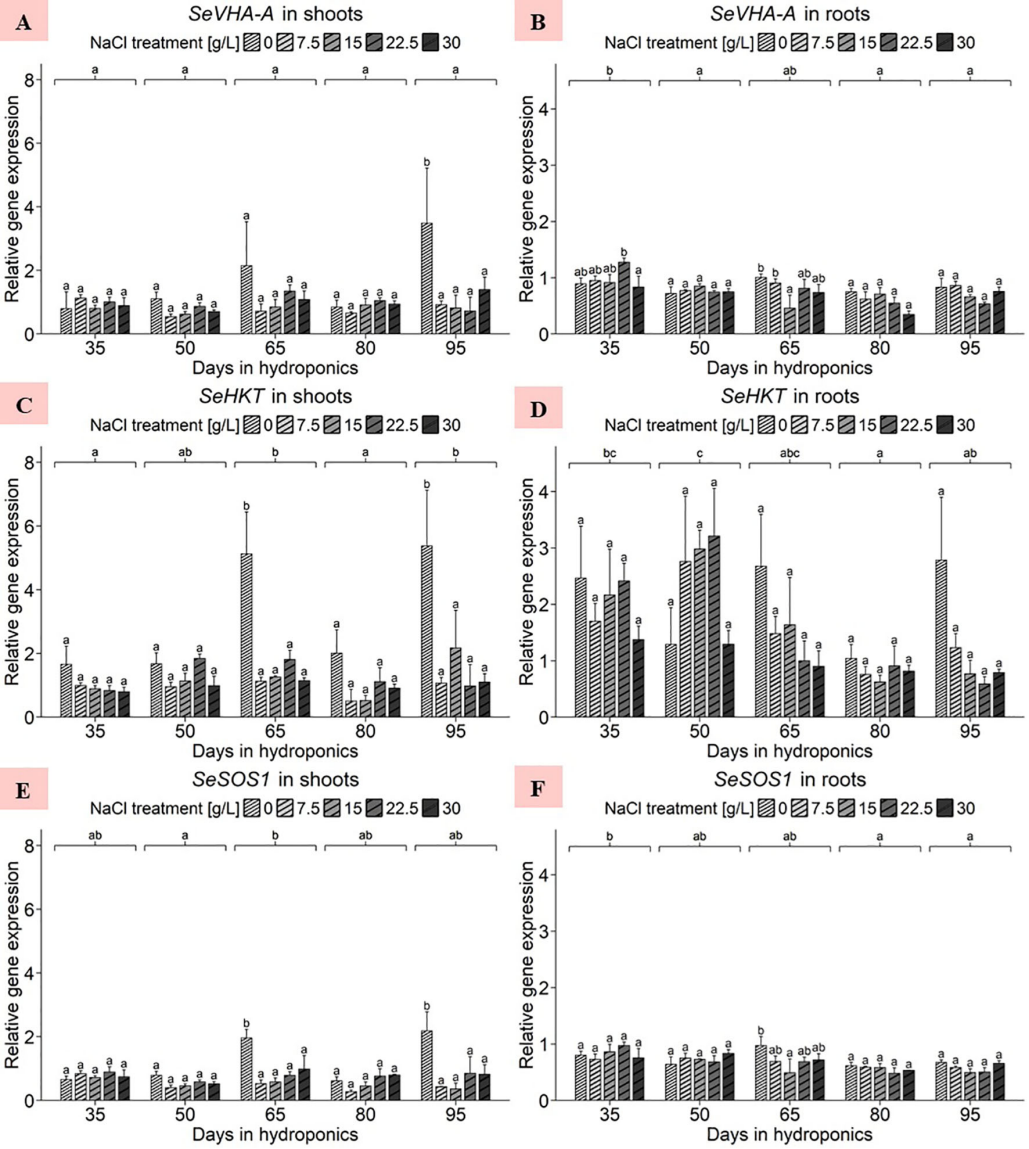
Gene expression analysis of *SeVHA-A*, *SeHKT* and *SeSOS1* in **(A, C, E)** shoots and **(B, D, F)** roots of *S. europaea* from various NaCl treatments (0, 7.5, 15, 22.5, and 30 g/L; equals 0, 128, 257, 385, and 513 mM) and developmental stages (hydroponics for 35, 50, 65, 80 and 90 d). Expression levels were normalized to the mean *SeUBC*, *SeActin* and *SeDNAJ* reference gene expression levels and RNA concentrations. The graphs show the means ± SEs of three replicates (74 plants in total; N.A. for one plant in the 30 g/L (= 513 mM) NaCl treatment in the fourth harvest group). Similar letters denote no significant difference (P value > 0.05).

Considering the shoot data, the ANOVA revealed no significant effects of the NaCl concentration, harvest group or their interaction term on the gene expression of *SeVHA-A* (**Supplementary Table 3**). Notably, in **Figure 5A**, the gene expression of plants treated with 0 g/L NaCl for 95 d increased, with an average significantly greater expression of 3.48 relative units compared to that of plants subjected to higher NaCl treatments in the same harvest group. A comparison of the mean expression levels in the NaCl treatments averaged across the five harvests revealed that the gene expression was predominantly elevated at 0 g/L NaCl, with an average of approximately 1.67 relative units (**Supplementary Table 4**). Additionally, the expression levels of *SeVHA-A* in the shoots were slightly greater in the 22.5 and 30 g/L NaCl treatment groups than in the 7.5 and 15 g/L NaCl treatment groups. For the root data, the ANOVA revealed significant effects of harvest group, none for NaCl treatment, but again significant effects of the interaction term of both variables on *S. europaea SeVHA-A* expression levels (**Supplementary Table 3**). Within individual harvests, shoot expression levels remained relatively consistent, as shown in **Figure 5B**. A comparison of the expression levels of the harvest groups averaged across the NaCl treatments indicated that the plants cultivated for 35 d had the highest overall gene expression levels, averaging 0.97 relative units, while the mean expression levels of the other harvests ranged from 0.59 to 0.78 relative units. However, when considering NaCl treatment effects with data averaged across harvests a downward trend of *SeVHA-A* expression with increasing NaCl levels was found (**Supplementary Table 4**).

For the shoots, the ANOVA revealed significant effects of harvest group, NaCl treatment, and their interaction term on the *S. europaea* gene expression of *SeHKT* (**Supplementary Table 3**). As presented in **Figure 5C**, after cultivation for 65 and 90 d in 0 g/L NaCl, *S. europaea* shoots showed a significant increase in gene expression compared to that in plants in the elevated NaCl treatment groups. The mean NaCl treatment effects were averaged across harvest groups and the expression range was set between 3.17 and 0.99 relative units at 0 g/L NaCl and 30 g/L NaCl, respectively (**Supplementary Table 4**). For the roots, the ANOVA revealed a significant effect of harvest group but not of NaCl treatment or the interaction term of both variables on *SeHKT* gene expression (**Supplementary Table 3**). As shown in **Figure 5D**, with the exception of harvest after 50 d of cultivation, *SeHKT* expression in each harvest group was highest in the roots of plants treated with 0 g/L NaCl. When averaged across all NaCl treatments, a cultivation period of 50 d resulted in the highest average expression of 2.31 relative units, while the roots of plants cultivated for 80 d displayed a significantly lower mean *SeHKT* gene expression of 0.83 relative units. Roots under 0 g/L and 30 g/L NaCl exhibited average expression levels of 2.05 and 1.04 relative units, respectively, while those under moderate NaCl treatment exhibited intermediate gene expression levels (**Supplementary Table 4**).

In the shoot samples, the ANOVA revealed significant effects of harvest group, NaCl concentration and their interaction term on *SeSOS1* gene expression (**Supplementary Table 3**). As shown in **Figure 5E**, cultivation for 65 and 95 d in 0 g/L NaCl significantly increased shoot *SeSOS1* expression, averaging 1.96 and 2.18 relative units, respectively. The mean *SeSOS1* expression in shoots averaged across harvest groups peaked at 0 g/L NaCl with 1.24 relative units, whereas the lowest average expression levels were found in shoots of the 7.5 and 15 g/L NaCl treatments with 0.49 and 0.52 relative units, respectively (**Supplementary Table 4**). With respect to the root samples, the ANOVA results indicated that the harvest group significantly affected *SeSOS1* expression, while the NaCl treatment and its interaction with the harvest group did not (**Supplementary Table 3**). **Figure 5F** shows that the expression of SeSOS1 increased in the roots of plants cultivated for 65 d at 0 g/L NaCl (0.97 relative units), which was significantly greater than that in the roots of plants treated with 15 g/L NaCl. Averaging the data across NaCl treatments, roots of *S. europaea* after cultivation for 35 d exhibited significantly increased gene expression levels of approximately 0.82 relative units compared to plants harvested after 80 and 95 d. Overall, the expression tended to decrease with increasing development. The analysis highlights the effects of NaCl treatments on roots averaged across harvest groups, where *SeSOS1* expression was most pronounced at 0 g/L NaCl, averaging 0.74 relative units (**Supplementary Table 4**). The remaining NaCl treatments resulted in average expression levels between 0.63 and 0.70 relative units.

#### Gene expression of *SePerox*, *SeAAP* and *SeVinS* in differently developed shoots and roots of *S. europaea* in response to various salt concentrations

**Figure 6:**
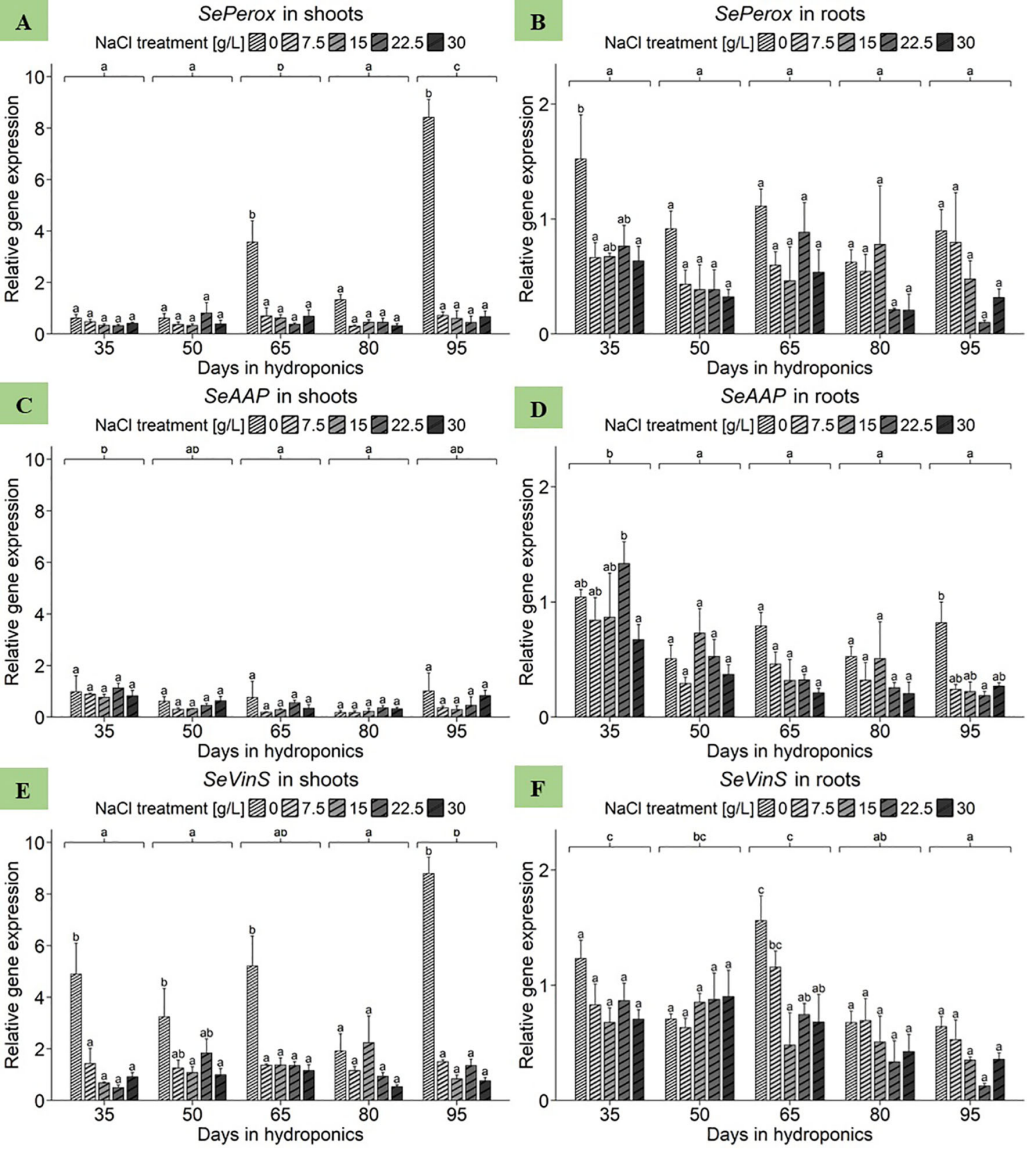
Gene expression analysis of *SePerox*, *SeAAP* and *SeVinS* in **(A, C, E)** shoots and **(B, D, F)** roots of *S. europaea* from various NaCl treatments (0, 7.5, 15, 22.5, and 30 g/L; equals 0, 128, 257, 385, and 513 mM) and developmental stages (hydroponics for 35, 50, 65, 80 and 90 d). Expression levels were normalized to the mean *SeUBC*, *SeActin* and *SeDNAJ* reference gene expression levels and RNA concentrations. The graphs show the means ± SEs of three replicates (74 plants in total; N.A. for one plant in the 30 g/L (= 513 mM) NaCl treatment in the fourth harvest group). Similar letters denote no significant difference (P value > 0.05).

With respect to the shoots of *S. europaea*, the ANOVA revealed significant effects of both harvest group and NaCl treatment as well as their interaction term on *SePerox* gene expression (**Supplementary Table 3**). Strikingly, as shown in **Figure 6A**, the 0 g/L NaCl treatment had the highest gene expression levels compared to the elevated NaCl treatments within each harvest group, with one exception being the 50 d harvest group. Analyzing NaCl effects by averaging expression data across all harvest groups, revealed shoot sensitivity to deficient salinity levels, with the expression being most pronounced at 0 g/L NaCl, with a mean of 2.91 relative units (**Supplementary Table 4**). In contrast, the gene expression levels of *SePerox* at elevated NaCl concentrations were consistently lower, between 0.47 and 0.51 relative units. With respect to the roots of *S. europaea*, the ANOVA highlighted the significant effects of both harvest group and NaCl treatment on the gene expression of *SePerox*, while the effects of their interaction term remained nonsignificant (**Supplementary Table 3**). In comparison to shoot samples, **Figure 6B** shows that the roots of *S. europaea* treated with 0 g/L NaCl again consistently exhibited the greatest gene expression, with the exception of those harvested after 80 d of cultivation. Moreover, this effect was more pronounced in the roots at the beginning of plant development, while it tended to occur in the shoots at mid to late development. When comparing mean expression levels between harvest groups without NaCl treatment effects, the roots of plants cultivated for 35 d had the highest expression levels, averaging approximately 0.85 relative units. Disregarding harvest group effects, roots showed an enhanced response to low salinity, with gene expression levels significantly peaking at 0 g/L NaCl at 1.01 relative units, while increasing NaCl levels resulted in a continuous decrease in mean *SePerox* expression (**Supplementary Table 4**).

First, focusing on shoot samples of *S. europaea*, the ANOVA revealed significant effects of harvest group on *SeAAP* expression (**Supplementary Table 3**). However, neither the NaCl treatment nor its interaction with harvest group significantly influenced the expression in *S. europaea* shoots. As presented in **Figure 6C**, the salinity-dependent expression profiles of the harvest group-specific genes varied without significant effects. Disregarding the effects of NaCl treatment, the shoots of plants cultivated for 35 d presented the greatest *SeAAP* gene expression, reaching a mean of 0.92 relative units. Conversely, the expression levels of genes in the shoots of *S. europaea* cultivated for 65 and 80 d were significantly lower, at 0.43 and 0.25 relative units, respectively. An analysis of the average salinity responses of shoot *SeAAP* expression across harvest groups revealed that the highest mean expression value was observed in the 0 g/L NaCl treatment group, with an average of 0.71 relative units, while the lowest was recorded in the 7.5 g/L and 15 g/L NaCl treatment groups (**Supplementary Table 4**). With respect to the roots, the ANOVA revealed significant effects of both harvest group and NaCl treatment on *SeAAP* gene expression (**Supplementary Table 3**). However, their interaction term was not significant. According to **Figure 6D**, with the exception of plants cultivated for 50 d, root *SeAAP* expression was highest in the 0 g/L NaCl treatment group of each harvest group. When averaging expression data across NaCl treatments for investigating harvest group effects, a significant increase in *SeAAP* expression was found in roots harvested after 35 d, with 0.95 relative units, whereas those cultivated for 95 d presented the lowest expression levels, averaging 0.35 relative units. Moving to the mean expression in roots in response to NaCl treatments averaged across harvest groups a peak in *SeAAP* expression at 0 g/L NaCl was observed, reaching a mean of 0.74 relative units (**Supplementary Table 4**). Conversely, gene expression in roots treated with 30 g/L NaCl was lowest, averaging 0.35 relative units.

Starting with the shoot data of *S. europaea*, the ANOVA revealed significant effects of harvest group, NaCl treatment and their interaction term on *SeVinS* expression (**Supplementary Table 3**). **Figure 6E** shows that after 35, 65 and 95 d of cultivation, shoot expression in the 0 g/L NaCl treatment group was significantly greater than that in the elevated NaCl treatment groups. Extending the view to analyze the effect of NaCl treatments averaged across harvest groups, it was evident that shoots displayed the highest average expression at 0 g/L NaCl, with a mean of 4.81 relative units, which consistently decreased until the lowest average expression was observed at 30 g/L NaCl, averaging 0.87 relative units (**Supplementary Table 4**). Considering *SeVinS* expression in *S. europaea* roots, the ANOVA revealed significant effects of the harvest groups and NaCl treatments but not of their interaction (**Supplementary Table 3**). **Figure 6F** revealed that, with the exception of the second harvest after 50 days, *SeVinS* expression levels were greater in shoots from the 0 and 7.5 g/L NaCl treatments than in those from the elevated NaCl treatments. Broadening the perspective by disregarding NaCl treatment effects to compare mean gene expression levels of harvest groups, expression in roots tended to decrease toward the end of the experiment. Furthermore, when studying NaCl treatment effects while disregarding variations in harvest groups, roots displayed a heightened sensitivity to lower NaCl concentrations (**Supplementary Table 4**). In detail, the gene expression of *SeVinS* was most pronounced at 0 g/L NaCl, with a mean of 0.96 relative units, while the roots of plants treated with 15 g/L NaCl exhibited a significantly lower average expression of the *SeVinS* gene, with a mean of 0.57 relative units.

#### Gene expression of *SeOsmP* and *SeProT* in differently developed shoots and roots of *S. europaea* in response to various salt concentrations

**Figure 7:**
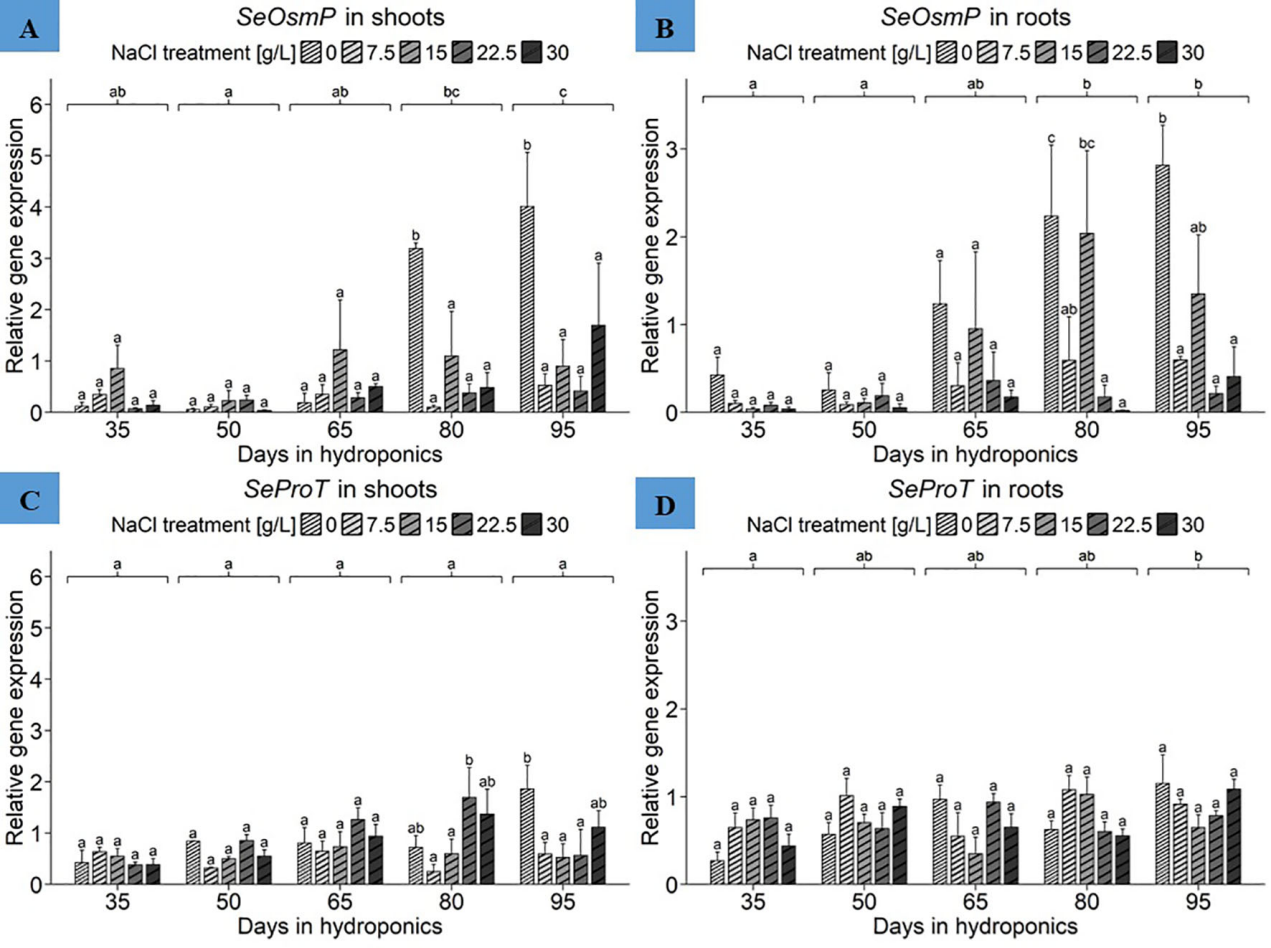
Gene expression analysis of *SeOsmP* and *SeProT* in **(A, C)** shoots and **(B, D)** roots of *S. europaea* from various NaCl treatments (0, 7.5, 15, 22.5, and 30 g/L; equals 0, 128, 257, 385, and 513 mM) and developmental stages (hydroponics for 35, 50, 65, 80 and 90 d). Expression levels were normalized to the mean *SeUBC*, *SeActin* and *SeDNAJ* reference gene expression levels and RNA concentrations. The graphs show the means ± SEs of the replicates (74 plants in total; N.A. for one plant in the 30 g/L (= 513 mM) NaCl treatment in the fourth harvest group). Similar letters denote no significant difference (P value > 0.05).

The ANOVA revealed significant effects of harvest group and NaCl treatment as well as their interaction term on Se*OsmP* gene expression in the shoots of *S. europaea* (**Supplementary Table 3**). As shown in **Figure 7A**, significantly increased expression levels in shoots were observed in plants cultivated for 80 and 95 d with 0 g/L NaCl, reaching 3.19 and 4.01 relative units, respectively. When averaged across NaCl treatments to reveal the mean expression levels of the harvest groups, plants cultivated for 95 d had the highest mean expression of approximately 1.51 relative units. The shoot highest mean *SeOsmP* expression levels were found in plants treated with 0 g/L NaCl, averaging 1.51 relative units, while 15 g/L NaCl led to the second-highest mean gene expression levels, with 0.86 relative units (**Supplementary Table 4**). According to the Se*OsmP* expression in the roots, the ANOVA revealed significant effects of both harvest group and NaCl treatment on Se*OsmP* gene expression in roots of *S. europaea*, but no significant effects were detected for the interaction term (**Supplementary Table 3**). **Figure 7B** shows that the roots of plants cultivated for 80 and 95 d with 0 g/L NaCl again exhibited mean Se*OsmP* expression levels of 2.23 and 2.81 relative units, respectively. During both harvests, compared with the 7.5, 22.5 and 30 g/L NaCl treatments, the 0 g/L NaCl treatment led to significantly increased gene expression levels in the roots of *S. europaea*. Similarly, after cultivation for 35, 50 and 65 d, the 0 g/L NaCl treatment resulted in higher gene expression levels than did the elevated NaCl treatments. Furthermore, beginning in the middle of plant development, 15 g/L NaCl led to increased Se*OsmP* expression in roots. From a broader perspective, regardless of NaCl treatment effects, the comparison of mean Se*OsmP* expression in the harvest groups revealed a steady increase in mean gene expression levels until the end of the experiment. Overall, the results accentuated similar responses of both roots and shoots to salinity when the Se*OsmP* gene expression levels were averaged across the harvest groups to determine the effects of NaCl treatments (**Supplementary Table 4**). The mean expression in roots significantly peaked in the 0 g/L NaCl treatment group, with an average of 1.39 relative units, while the lowest value was detected in the 30 g/L NaCl treatment group, with an average of approximately 0.14 relative units. Additionally, plants treated with 15 g/L NaCl had elevated mean expression levels of the Se*OsmP* gene in roots, with 0.90 relative units.

Focusing initially on the shoots, the ANOVA results showed a significant influence of harvest group, NaCl treatment and their interaction term on *SeProT* gene expression in *S. europaea* (**Supplementary Table 3**). **Figure 7C** shows that the peak *SeProT* gene expression occurred in the shoots of plants cultivated for 95 d with 0 g/L NaCl, peaking at approximately 1.86 relative units, which was significantly greater than the corresponding gene expression levels in the 7.5, 15 and 22.5 g/L NaCl treatment groups. In previous harvests, gene expression in the 0 g/L NaCl treatment group was in the middle of the expression range found in the respective harvest group, while cultivation for 50, 65 and 80 d resulted in increased gene expression in the shoots of plants treated with 22.5 g/L NaCl. Disregarding NaCl treatment responses to compare means of *SeProT* gene expression between harvest groups, the lowest mean gene expression levels in shoots, approximately 0.48 relative units, were observed in early development represented by 35 d of cultivation. Moreover, there was a constant increase in *SeProT* gene expression from 35 d to 80 d of cultivation. The expression means of NaCl treatments disregarding harvest group variations highlighted the increase in shoot *SeProT* expression under 22.5 g/L NaCl conditions to an average of 0.95 relative units, followed by 0.93 relative units under 0 g/L NaCl conditions (**Supplementary Table 4**). For the root data, the ANOVA revealed significant effects of harvest group and their interaction term with NaCl treatment (**Supplementary Table 3**). As presented in **Figure 7D**, the roots of plants cultivated for 95 d in the 0 g/L NaCl treatment showed increased *SeProT* expression, reaching 1.15 relative units. In contrast, after cultivation for 35 d with 0 g/L NaCl, the overall lowest expression levels in roots, with 0.27 relative units, were recorded. By expanding the frame to harvest group effects while disregarding NaCl treatments, cultivation for 95 d resulted in increased mean expression levels of 0.92 relative units, while roots cultivated for 35 d exhibited the lowest gene expression at 0.57 relative units. With the exception of the harvest after 50 d, a constant increase in *SeProT* expression levels was observed with the ongoing development of *S. europaea*. Presenting NaCl treatment responses of plants disregarding harvest groups, elevated mean *SeProT* expression levels were detected in the roots of plants in the 7.5 g/L NaCl treatment group, with an average of 0.84 relative units (**Supplementary Table 4**).

## Discussion

### Differential physiological responses to sub- and superoptimal salinities in *S. europaea*: variations in growth reduction causes despite comparable growth impairments

Fresh weight measurements indicated plant growth under different NaCl conditions (**Figure 1**). Previous studies on *S. bigelovii* showed different physiological responses to suboptimal (5 mM NaCl) and superoptimal (600 mM NaCl) treatments (Ayala and O'Leary, 1995). In the present investigation, both salt deficiency and excess reduced growth of *S. europaea*, with salt deficiency (0 g/L NaCl) having a more pronounced effect than salt excess (30 g/L NaCl). The increase in shoot weight towards the experiment’s end likely relates to reduced competition for space, indicating plant density’s importance in optimizing *S. europaea* cultivation regardless of salinity levels.

High salinity can hinder halophyte growth due to mechanisms such as reduced CO_2_ capture for photosynthesis, disrupted energy balance, improper resource allocation, disturbed water balance, and increased energy needs for water management (Flowers and Colmer, 2008). Reduced shoot biomass in superoptimal salt treatments is likely due to these factors.

Potassium (K^+^) is essential for plant life and likely fulfills similar metabolic roles in both halophytes and glycophytes. This is supported by similar cytoplasmic K^+^ concentrations observed in actively growing halophyte cells compared to glycophytes (Gorham and Wyn Jones, 1983; Flowers et al., 1986). Plants in saline environments have evolved mechanisms for selective K^+^ uptake despite the abundance of Na^+^ (Maathuis and Amtmann, 1999). However, halophytes also use Na^+^ for osmotic adjustment, requiring them to select K^+^ from a Na^+^-dominated mixture and still accumulate sufficient Na^+^ for osmotic adjustment. Studies have shown that media lacking or containing only low Na^+^ (e.g., 10 mM) increases K^+^ accumulation in halophytes, consequently impairing growth of various halophytes (Ashby and Beadle, 1957; Williams, 1960; Mozafar et al., 1970; Yeo and Flowers, 1980; Matoh et al., 1986; Wang et al., 2001). Yeo (1981) proposed, based on flux analysis data, that halophytes like *S. maritima* can sequester Na^+^ within vacuoles, but not K^+^. Additionally, *S. maritima* grown under Na^+^ deficiency exhibited respiration rates about twice as high compared to those grown in moderate to high salinity (Flowers, 1972). These observations could be linked to the presumed low permeability of halophyte tonoplasts (vacuolar membranes) to Na^+^ and Cl^-^. If true, replacing these ions with other solutes like K^+^ for osmotic regulation becomes energetically expensive. This suggests that halophytes like *S. maritima* experiencing salt deficiency must dedicate more energy towards osmoregulation, potentially leading to a subsequent reduction in growth. Conformingly, previous research suggests that sodium plays a more critical role than potassium and chloride in promoting various aspects of *S. europaea* vitality (Lv et al., 2012b). Furthermore, studies on *S. bigelovii* showed that the relative growth rate (RGR) continuously decreased but the photosynthetic rate increased despite lower water use efficiency under suboptimal NaCl conditions (5 mM NaCl) during the 40-day growth phase (Ayala and O'Leary, 1995). Under suboptimal NaCl supply, increased uptake of K^+^, Ca_2_^+^, and Mg_2_^+^ attempted to compensate for Na^+^ deficiency, potentially contributing to growth reduction through toxic effects.

Regarding the darker appearance of shoots grown under suboptimal salinity in the present study, previous research has shown that in young plants of *Salicornia persica*, *Salicornia bigelovii* and *S. europaea*, the chlorophyll a, chlorophyll b and carotenoid contents were approximately twice as high after 8 days of treatment with 0 mM NaCl compared to those after treatment with 500 mM NaCl (Homayouni et al., 2024). Quite similar, a steady decrease in chlorophyll a, chlorophyll b and carotenoids with increasing salinity was observed in seedlings of *S. persica* and *S. europaea* treated with seven concentrations between 0 and 600 mM NaCl (Aghaleh et al., 2009). Further research on *S. brachiata* revealed that under conditions of 100% seawater (32.5 ppt) and 0.5 M NaCl salinity, plants exhibited comparatively greater gas exchange, stomatal conductance, and PSII photochemistry than did plants cultivated in tap water (Siddiqui et al., 2022).

### Salinity differentially affects levels of total phenolic compounds in shoots and roots of *S. europaea*

As shown in **Figure 2**, salinity significantly affects phenolic compound accumulation primarily in the shoots of *S. europaea*, while the developmental stage significantly influences phenolic content in roots. A previous study found no significant effects of NaCl treatments (0.1 - 769 mM NaCl) on phenolic content in two genotypes of *Salicornia neei* Lag. after 50 days of cultivation (Souza et al., 2018). Similarly, no major differences in total phenol content were observed in the shoots of *S. ramosissima* when comparing a 30-day optimal salt treatment with a superoptimal salt treatment (optimal 171 mM NaCl; superoptimal 510 mM NaCl) (Mesa-Marín et al., 2023). As previously discussed regarding the toxicity of accumulating ions other than Na^+^ under prolonged NaCl deficiency in *Salicornia dolichostachya* (Katschnig et al., 2013), 0 g/L NaCl is insufficient to sustain the growth of various *Salicornia* species. The stress reaction caused by NaCl deprivation appears to intensify in shoots during later developmental stages, and is likewise reflected in increasing phenolic compound concentrations. The continuous, salt-independent increase in root phenolic compounds with plant age, unaffected by salinity changes, suggests a distinct regulatory mechanism.

### Gene expression analysis – need for adaptation of reference gene selection to experimental settings

Previous research revealed that the reference genes *SeCAC* and *SeUBC* under NaCl stress, *SeTIP41* and *SeDNAJ* under heat stress, *SeUBC* and *SeUBQ* for different tissues and *SeUBC* and *SeActin* at different developmental stages are most suitable for normalizing gene expression levels in *S. europaea* (Xiao et al., 2015). Based on lower amplification quality of *SeCAC* in shoot samples, the gene was assessed as less suitable for normalization. Similarly, *SeUBQ* was discarded due to its relatively high variance in root samples (**Figure 3**). Within the more detailed evaluation of reference gene suitability, *SeTIP41* was found less suitable in certain data subsets (**Supplementary Figure 2**). Therefore, the best reference gene candidates to ensure the comparability of organ-specific and salinity-affected expression levels for the genes of interest were *SeUBC*, *SeActin* and *SeDNAJ*.

### Gene expression analysis - the challenges of comparability

To gain new insights into long-term adaptive gene regulation in *S. europaea*, gene expression responses to salinity conditions at different developmental stages will be examined. The transcriptional patterns in plants already adapted to salinity fundamentally differ from short-term reactions to sudden changes in salinity, which often complicated direct comparisons with previous research findings. The following discussion will move beyond the temporally narrow focus of earlier investigations that primarily analyzed immediate post-treatment responses.

#### Tissue-dependent regulation of genes related to sodium transport chain, including *SeNHX1*, *SeVP1*, *SeVP2*, *SeVHA-A*, *SeHKT* and *SeSOS1*, is strongly affected by salt deficiency

The electrochemical H^+^ gradient created by V-H^+^-ATPase (*SeVHA-A*) and V-H^+^-PPase (*SeVP1* and *SeVP2*) across the tonoplast tightly regulates the Na^+^/H^+^ antiporter (*SeNHX1*) activity (Rea and Poole, 1993). Overexpression of *NHX1* from *Salicornia brachiata* in tobacco enhances salt tolerance, highlighting antiporters’ role in ionic homeostasis (Jha et al., 2011). Na^+^ accumulation under NaCl treatment correlates with increased transcription of *SeNHX1*, *SeVHA-A*, and *SeVP1*, while *SeVP2* remains unaffected by salinity (Lv et al., 2012a). This indicates that the expression of Na^+^/H^+^ antiporter, V-H^+^-ATPase, and at least one V-H^+^-PPase gene closely responds to Na^+^ influx, activating vacuolar translocation mechanisms. Further research revealed that vacuolar Na^+^ sequestration depends on V-H^+^-ATPase and V-H^+^-PPase activities, with *SeVHA-A* expression influencing V-H^+^-PPase and Na^+^/H^+^-antiporter gene expression in *S. europaea* (Lv et al., 2017). **Figures 4A, B** shows that *S. europaea* shoots exhibit heightened sensitivity to sodium deprivation, which could be considered a stress-inducing nutrient deficiency in obligate halophytes. Increased Na^+^/H^+^-antiporters at the tonoplast may prepare the plant for salt deficiency, with transcript levels particularly influenced at later developmental stages, suggesting intensified gene regulatory response due to persistent NaCl shortage. In contrast, *SeNHX1* expression in roots suggests its role in short-term buffering of high salinity by vacuolar Na^+^ sequestration in root tissues (Jha et al., 2011). These findings indicate an additional buffering function to roots in *S. europaea*. The increased reactivity of shoots to salt deprivation implies that long-term compensatory mechanisms to mitigate salt deficiency toxicity are more prominent in shoots than in roots. Excessive salt in roots, requiring translocation to shoots for storage, may lead to intermediate vacuolar storage in root cells. In contrast, salt deficiency in shoots stimulates Na^+^/H^+^ antiporter production, aiding in pumping insufficient Na^+^ into vacuoles to maintain internal cell pressure and Na^+^-dependent osmoregulation.

The long-term salt-deprived responses of *SeVP1* and *SeVP2* (**Figures 4C–F**) contrast with previous findings of short-term upregulation under elevated salinity (Lv et al., 2012a, 2015). Low salt concentrations may negatively affect the H^+^-gradient between the vacuole and cytoplasm over the long term, necessitating constant upregulation of both V-H^+^-PPase genes in shoots under persistent 0 g/L NaCl conditions. This is supported by the increasing expression levels at later stages of development, indicating elevated sodium-deprived stress from a prolonged lack of Na^+^ ions. Additionally, *SeVP2*’s response to salt deficiency is less pronounced, suggesting that in *S. europaea*, the H^+^-gradient in photosynthetically active organs relies more on *SeVP1*’s activity, while both V-H^+^-PPases in roots are regulated similarly, regardless of salinity variation.

V-H^+^-ATPase (SeVHA-A), another key component in the Na^+^ sequestration system, also contributes to the electrochemical H^+^-gradient at the tonoplast. Thus, the gene expression patterns of *SeVP1*, *SeVP2*, and *SeVHA-A* show little difference in response to salinity. The increased expression of *SeVHA-A* in both low- and high-NaCl treatment groups can be attributed to stress from sodium deficiency and excessive salinity. Previous studies showed significant upregulation of *SeVHA-A* in response to elevated NaCl levels in shoots (Lv et al., 2012a, 2017). While these studies emphasize *SeVHA-A*’s role in high-salinity tolerance, the present findings suggest nuanced modulation of *SeVHA-A* expression in salinity-adapted plants, with upregulation in shoot tissues due to nutritional salt-deprivation stress.

Continuing within the transporter gene group, **Figures 5C, D** indicates heightened sensitivity to sodium scarcity during certain developmental periods. The roots of *S. europaea* exhibited decreasing expression of *SeHKT* across various harvest groups, while the shoot expression increased with development, suggesting reciprocal regulation of sodium transport between the tissues. The exact functions of various *HKT* transporters remain unclear (Ma et al., 2013). The diverse roles of *HKT* genes in salinity adaptation are evidenced by their variability in ion selectivity and transport mechanisms among different family members (Hamamoto et al., 2015; Su et al., 2015). For instance, *SeHKT1* in *S. europaea* is particularly responsive to excessive NaCl levels in roots, indicating a critical role in ion uptake and homeostasis (Ma et al., 2015). The absence of *HKT1;1* expression in *S. dolichostachya* suggests that not all *Salicornia* species utilize the same *HKT* genes or pathways for salinity tolerance (Katschnig et al., 2015). This study suggested that high Na^+^ accumulation in *S. dolichostachya* shoots could be due to increased *SOS1*-mediated Na^+^ loading in the xylem of mature roots. Conversely, *SeHKT1;2* expression in *S. europaea* significantly increased in shoots with 10 mM NaCl but not with higher concentrations (200 mM, 500 mM, 800 mM) of NaCl (Haxim et al., 2023). The roots gradually decreased in expression with increasing NaCl concentration, aligning with the present results in **Figures 5C, D**. Further, the reaction to salinity in shoots aligns with previous findings where *SeHKT*, homologous to a hkt1-like gene in *Populus trichocarpa*, was upregulated with increasing salinity but decreased when NaCl exceeded 200 mM (Ma et al., 2013). Consistent with the present study, *SeHKT* was downregulated in roots under excessive salinity (30 g/L). Overall, *SeHKT* expression patterns in *S. europaea* reflect dynamic, organ-specific, and species-specific adaptations to salinity, with transcript levels increasing in roots at early growth stages and in shoots mainly under NaCl deficiency.

In root systems, Na^+^ translocation into the xylem involves the *SOS1-*encoded Na^+^/H^+^ antiporter (Shi et al., 2002). In some instances, passive Na^+^ loading may occur through NORC channels (Wegner et al., 2011). The *SOS1* transporter, located in the plasma membrane, transports Na^+^ from the cytoplasm to the extracellular space and is regulated by the SOS pathway, which also influences some vacuolar ion transporters (Oh et al., 2010). *SOS1* expression is predominantly observed in epidermal cells at root tips and in cells adjacent to the root and shoot vasculature (Oh et al., 2009). In the present study, shoot tissues from plants adapted to near-optimal salt concentrations (15 g/L NaCl) displayed lower *SeSOS1* gene expression compared to those under extreme NaCl concentrations (0 g/L and 30 g/L NaCl) (**Figure 5E**). The upregulation of *SeSOS1* gene expression in shoots during later development suggests that persistent sodium deprivation leads to increasing nutrient deficiency effects in *S. europaea*.

In summary, the increased transcript levels in shoots at 0 g/L NaCl indicate that their upregulation might promote the uptake of insufficient NaCl. The above-mentioned specialized transporters may not be ideally suited for the uptake of alternative ions such as Mg^2+^, Ca^2+^ and K^+^, necessitating high transcription rates to compensate for the lack of NaCl (Ayala and O'Leary, 1995). The continuously increased outflow of these alternative ions may also explain the increased need for transporters to pump the ions back into the leaky vacuoles. In contrast, if the salt concentration increases acutely, *Salicornia* reacts by increasing the expression of H^+^-transporters to maintain vacuolar acidity, facilitating the activity of Na^+^/H^+^ antiporters. Furthermore, *SeHKT* and *SeSOS1* play crucial roles in redistributing Na^+^ from roots to shoots. The tissue-dependent regulation patterns suggest a nutritional deficiency linked to the vital demand for NaCl in shoots. In *S. europaea* shoots, *SeSOS1* likely supports *SeHKT* transporters when salt is present in excess. *SeNHX1*, *SeVP1*, *SeVP2* and *SeHKT* are particularly suitable biomarkers in the shoots of *S. europaea* for detecting salt deficiency.

#### Gene expression of *SePerox*, *SeAAP* and *SeVinS* in differently developed shoots and roots of *S. europaea* in response to various salt concentrations

Peroxidases, distinct isoenzymes that can be constitutively transcribed or induced by external factors such as wounding, stress, and pathogen attack, have versatile antioxidant functions in plants (Pandey et al., 2017). The increasing transcription rate of *SePerox* during prolonged salt deficiency (**Figure 6A**) contrasts with previous research showing a significant increase in *SePerox* expression in close response to rising salinity (Ma et al., 2013). Further research demonstrated that enzymatic peroxidase activity in *S. europaea* is greater under salinity and drought stresses, highlighting its protective function (Torabi and Niknam, 2011). In the present study, prolonged salt deficiency might induce greater stress than a moderate excess of NaCl in *S. europaea*. These findings collectively suggest that *S. europaea* utilizes peroxidases as pivotal components in its stress response, particularly under salinity challenges. Overall, the results indicate that the transcription of *SePerox* might function as a stress-related biomarker induced by prolonged salt deficiency as well as salinity challenges in general.

In *A. thaliana*, the genes *AAP8* and *AAP1*, which encode amino acid permeases, are involved in supplying amino acids to seeds during the reproductive phase (Tegeder, 2012). This could explain the upregulation of *SeAAP* in *S. europaea* shoots at the end of the experiment, correlating with flower induction. In *S. brachiata*, the total content of free amino acids, proline, and polyphenols increased with drought stress (Parida and Jha, 2013), suggesting that the compounds help maintain osmotic balance and scavenge free radicals. Similar processes likely occur in *S. europaea* under salt deficiency stress to cope with a nutritional lack of Na^+^ ions. The expression pattern in roots supports the theory of upregulated *SeAAP* expression in response to Na^+^-deprived conditions. Thus, *SeAAP* expression can be considered a biomarker for detecting suboptimal and superoptimal salinity levels throughout *S. europaea*’s development, especially in shoots.

The results highlighted a decrease in Se*VinS* expression in *S. europaea* in shoots and roots under long-term salinity excess. This contrasts with the stable *vinorine synthase* expression observed in *Morus* spp. in response to salinity changes (Gan et al., 2021) (**Supplementary Table 4**). However, these findings align with the downregulation of a vinorine synthase-like gene in salt-stressed *Solanum tuberosum* L (Li et al., 2020), suggesting variable regulation across plant families. Consistent with previous reports (Ma et al., 2013), *SeVinS* was upregulated under salt-free conditions and downregulated under excessive NaCl in *S. europaea*. This pattern indicates that *SeVinS* could serve as a biomarker for detecting salt deficiency stress in plants.

#### Tissue- and developmental dependent osmoregulation related to *SeOsmP* and *SeProT* in *S. europaea* is primarily affected by long-term NaCl deficiency stress

Stress-protective proteins such as osmotin and osmotin-like proteins are crucial for plant adaptation to osmotic stress (Kosová et al., 2013). Increases in these proteins have been observed in salt-treated plants, including the halophytic mangrove *Bruguiera gymnorhiza* (Tada and Kashimura, 2009). The present findings, shown in **Figures 7A, B**, partially corroborate previous results (Ma et al., 2013), demonstrating upregulation of Se*OsmP* expression in response to both optimal and deficient salinity levels. However, no increase in *SeOsmP* expression was observed in roots under elevated salt concentrations (22.5 and 30 g/L NaCl), aligning with earlier reports. The results support the synthesis of low-molecular-weight compounds for osmoregulation under suboptimal salinity (Ma et al., 2013). Previous research, such as one where tobacco derived *osmotin protein* overexpression in soybean conferred salt tolerance (Subramanyam et al., 2012), suggest that increased gene expression under 0 g/L NaCl conditions aids in osmoregulation. Additionally, *SeOsmP* transcription increased with plant age in *S. europaea*, indicating a persistent nutritional deficiency due to long-term sodium deprivation.

Research has shown a correlation between salinity and proline content in *S. europaea*, with proline levels increasing at NaCl concentrations above 800 mM (Cárdenas-Pérez et al., 2021). Similarly, *S. brachiata* exhibited higher proline levels under 500 mM NaCl compared to 0 mM NaCl treatments (Benjamin et al., 2019). This suggests that *SeProT* upregulation might be detectable at salinity levels higher than 30 g/L NaCl in *S. europaea*. Moreover, previous findings also indicated *SeProT* upregulation in close response to salt treatments (Ma et al., 2013), although this was presently not detected in plants adapted to the prolonged salinity levels (**Supplementary Table 4**). This implies that *SeProT* upregulation in response to salt deficiency may require a longer time frame and does not occur immediately with short-term salt deficiency. The consistent upregulation of *SeProT* during plant development, as shown in **Figures 7C, D**, reflects an adaptive response essential for maintaining osmotic potential.

Notably, increased *SeOsmP* expression in shoots and roots under salt deficiency toward later development suggests its biomarker usability for sodium shortages. Similarly, in NaCl deficient conditions proline becomes increasingly important in shoots with plant age under NaCl deficiency, indicating that *SeProT* transcription in shoots might serve as a biomarker for unfavorable salinity levels.

## Conclusion

*Salicornia europaea* exhibits a broad spectrum of physiological responses to varying salinity levels, which can have positive or negative consequences for its growth and development. While high salinity can be detrimental, the lack of Na^+^ as an essential nutrient also disrupts its well-being. Notably, at 0 g/L NaCl, a unique set of genes in shoots showed increased gene expression levels, especially during mid to late development, highlighting a nutrient deficit in the long-term absence of Na^+^ ions. These genes included *SeNHX1*, *SeVP1*, *SeVP2*, *SeVHA-A*, *SeHKT*, *SeSOS1*, *SePerox* and *SeVinS*. *SeHKT* and *SeOsmP* showed significantly increased gene expression levels in the roots under salt deficiency conditions, with *SePerox* and *SeVinS* also showing signs of upregulation under these conditions at certain developmental stages. The expression levels of these genes can thus be regarded as biomarkers for a sodium nutritional deficiency in the corresponding tissues. The distinct expression patterns observed in shoots and roots may reflect evolutionary adaptations, optimizing the overall response of plants to fluctuating salinity levels encountered in their natural habitat, with differences between the species within Salicornioidaea reflecting nuanced adaptation strategies to their specific saline environments. The overall more pronounced expression responses of the analyzed genes especially to salt deficiency in shoots compared to roots suggest that shoot tissues have a stricter sodium requirement for maintaining essential physiological processes. Additionally, the findings show that *S. europaea* achieves its highest shoot fresh mass at 15 g/L NaCl, indicating an optimal sodium supply. In contrast, the lowest fresh mass was correlated with the highest levels of total phenolic compounds under 0 g/L NaCl, which, in conjunction with certain significantly increased gene expression levels, indicates increasing sodium deprived nutritional stress in *S. europaea*. Furthermore, the increase of sodium deprived reactions in mid to late development indicates a cumulative, negative effect of sodium deficiency. This could presumably be caused by the replacement of Na^+^ ions by alternative cations, which result in a significantly higher energy consumption to maintain the vital turgor. Deciphering the developmentally regulated gene expression patterns in *S. europaea* under conditions of nutritional sodium deficiency or sodium excess-induced toxicity holds significant value for optimizing agricultural practices in saline environments. Therefore, this investigation into the response of euhalophytes (obligate halophytes) to sodium deficiency reveals a previously underappreciated role for Na^+^ as an essential nutrient. Even if it could be inferred from the definition of the word obligatory, severe sodium deficiency does not cause immediate growth inhibition or death, but rather exhibits a progressive negative impact on plants growth over time. This special nutritional deficiency triggers multiple stress and defense reactions in euhalophytes. These findings broaden our understanding of the diverse physiological responses in halophytic plants and challenge the previous focus solely on their tolerance to excess salinity. Notably, sodium deficiency should be recognized as a nutritional stress only in euhalophytes, as optimal growth depends on adequate sodium availability. This knowledge can be leveraged to cultivate novel halophytic crops or engineer salt-tolerant cultivars of existing crops, employing both traditional breeding programs and advanced biotechnological tools. Deciphering developmental processes in halophytes can empower researchers to recommend optimal harvest timing for maximizing both yield and the production of health-promoting secondary metabolites in saline agriculture. This knowledge can translate into practical applications for farmers including strategies like salinity regulation through freshwater irrigation. Soils with varying salinity levels could become attractive for cultivating halophytes, such as *S. europaea*. Furthermore, research into the diverse compositions of saline soils holds promise for establishing plant cultures for a wide range of salt-adapted plants.

## Data Availability

Main data generated or analyzed during this study are included in this published article and its **Supplementary Materials**. Main raw data is stored in an additional file (**Supplementary Table 5**). Further materials used in the present study are available under an MTA from the corresponding author upon reasonable request.
