# Supplementary material for: Molecular analysis of the reactions in Salicornia europaea to varying NaCl concentrations at various stages of development to better exploit its potential as a new crop plant
Source: Front Plant Sci. 2024 Sep 3;15:1454541. doi: 10.3389/fpls.2024.1454541 (PMC11405239; doi:10.3389/fpls.2024.1454541)
Supplement: Supplementary file 1 [file DataSheet1.zip › Supplementary Figure 1.pdf]

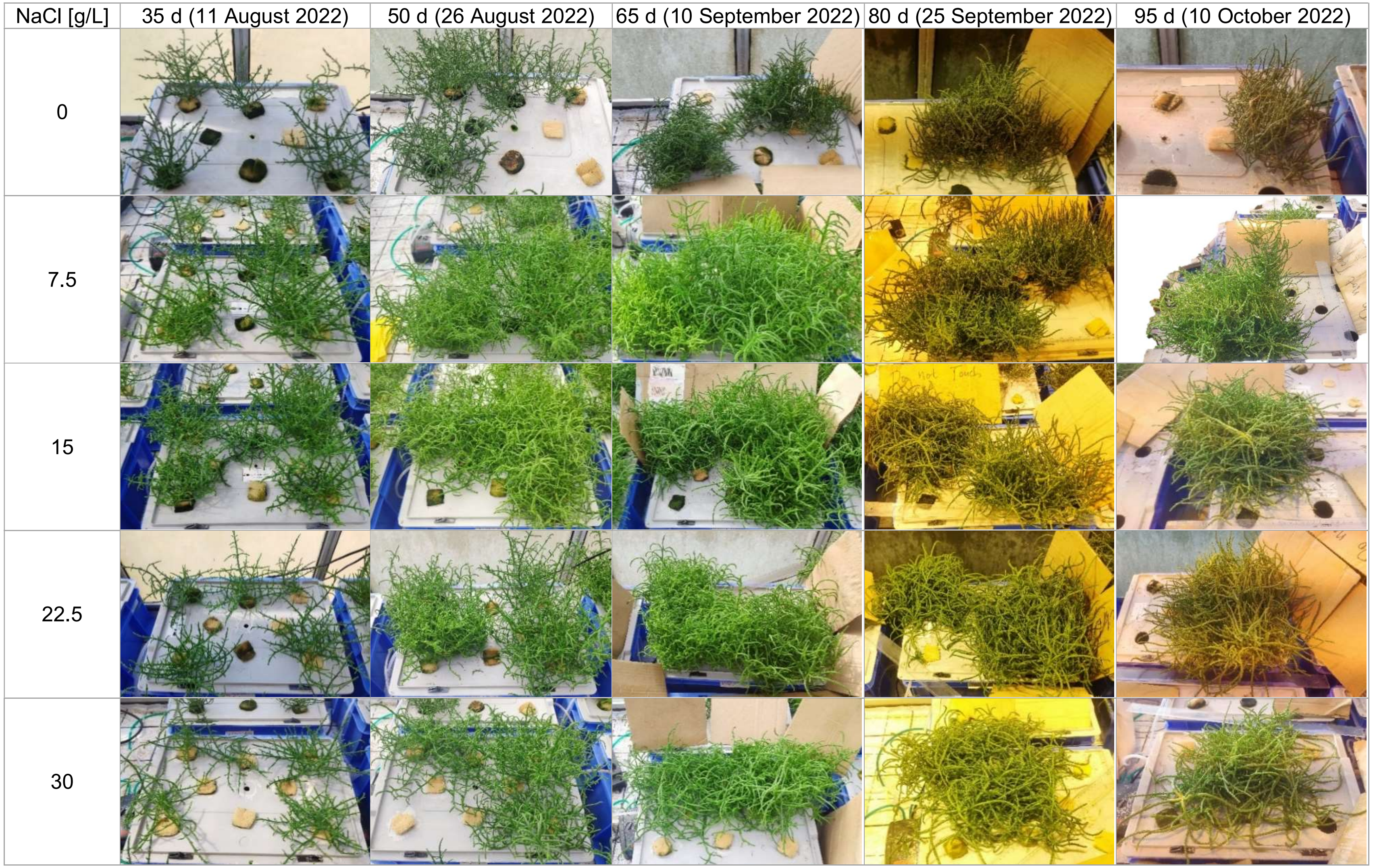

Supplementary Figure 1. Representative images of *S. europaea* plants growth in Hoagland media with varied NaCl concentrations [0-30 g/L] in hydroponics with images taken before harvests after 35, 50, 65, 80 and 95 d.
