# Supplementary material for: Molecular analysis of the reactions in Salicornia europaea to varying NaCl concentrations at various stages of development to better exploit its potential as a new crop plant
Source: Front Plant Sci. 2024 Sep 3;15:1454541. doi: 10.3389/fpls.2024.1454541 (PMC11405239; doi:10.3389/fpls.2024.1454541)
Supplement: Supplementary file 1 [file DataSheet1.zip › Supplementary Figure 2.pdf]

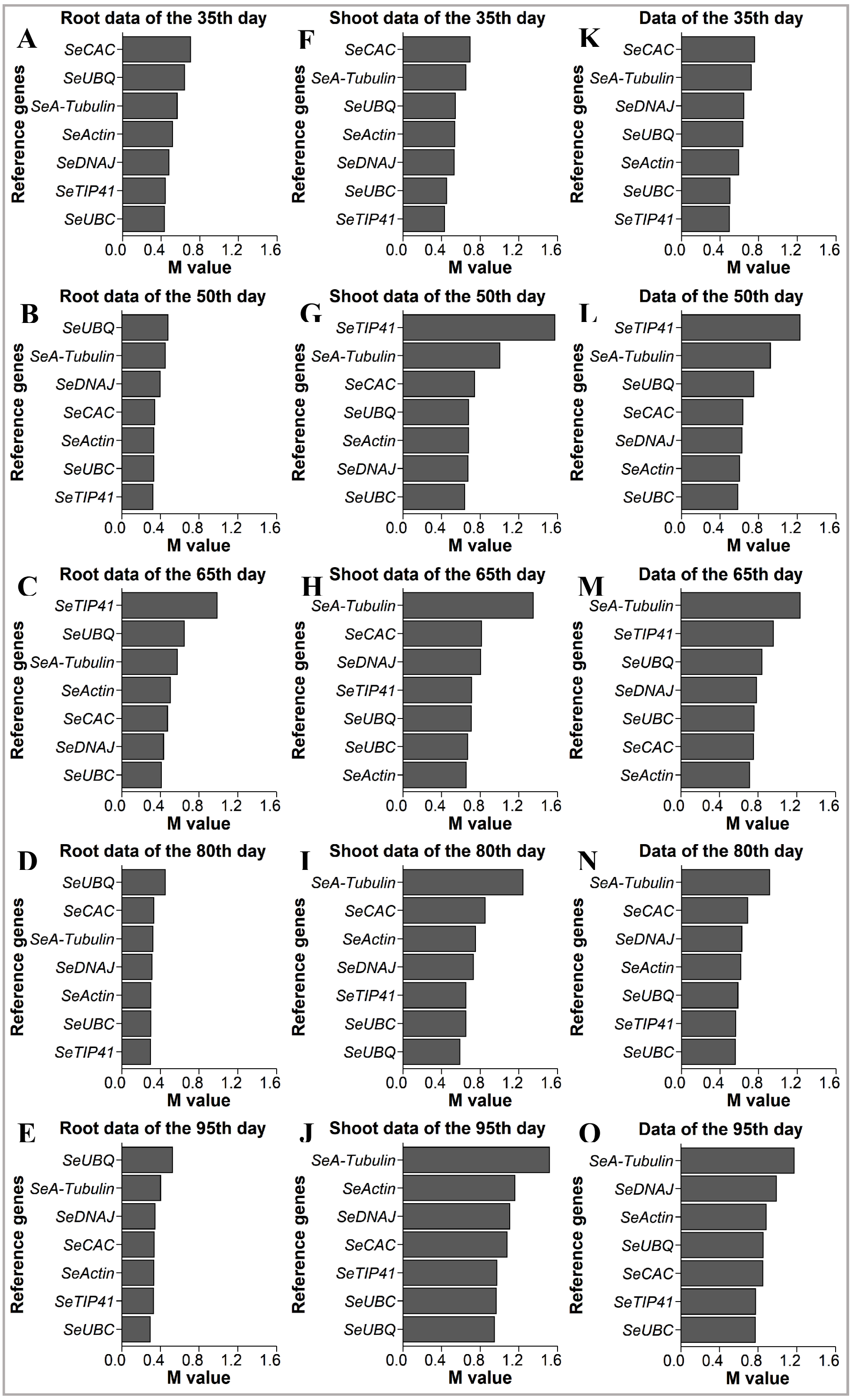

Supplementary Figure 2. geNorm M values with smaller values indicating higher stability and related genes as better options for normalization of qPCR data for expression levels of indicated candidate genes in *S. europaea* **(A-E)** root samples, **(F-J)** shoot samples and **(K-O)** total samples, of individual harvests after 35, 50, 65, 80 or 95 d of hydroponic cultivation. Expression was normalized using RNA concentrations (74 plants in total; N.A. for one plant of 30 g/L NaCl treatment in fourth harvest group).
