# Supplementary material for: Molecular analysis of the reactions in Salicornia europaea to varying NaCl concentrations at various stages of development to better exploit its potential as a new crop plant
Source: Front Plant Sci. 2024 Sep 3;15:1454541. doi: 10.3389/fpls.2024.1454541 (PMC11405239; doi:10.3389/fpls.2024.1454541)
Supplement: Supplementary file 1 [file DataSheet1.zip › Supplementary Table 1.pdf]

Supplementary Table 1. Primer sets, genes, primer sequences, gene/-functions and cellular functions of reference genes and genes of interest for S. *europaea* with sequence information for designing primers as well as gene functionalities/annotations modified after Lv et al. 2012a[X], Ma et al. 2013[Y] and Xiao et al. 2015[Z] (X,Y and Z indicate the respective assignment of the original sequence information and functional annotation to the three publications mentioned).

Primer set	Gene acronym	Forward Primer (5‘ – 3‘)	Reverse Primer (3‘ – 5‘)	Gene or gene function	Cellular function
P1[Z]	*Seα-tub*	GAGATCACCACCAGTGCCTTTG	GTGGCCACAGCAGCGTTCAC	α-Tubulin subunit related	Formation of microtubules as part of the cytoskeleton
P2[Z]	*SeActin*	GCACGGTATTGTGAGTAACTGGG	CCTGTTAGCCTTAGGGTTCAATG	Actin related	Actin is a structural protein and part of the cytoskeleton
P3[Z]	*SeDNAJ*	CCCAGCAAGAGGCTTACGAAG	CCTTCCATCTTTTGCGCCTATCAC	Chaperone DnaJ protein related	Heat shock protein (Hsp) family; protect proteins from irreversible aggregation during synthesis/ cellular stress
P4[Z]	*SeUBC*	CTGTAGTGCAGGGCCAGTCG	GAATAGATACGAGAAATACACCTCCC	Ubiquitin C related	DNA repair, endocytosis, stress response and signal transduction
P5[Z]	*SeUBQ*	GACCCTTACAGGAAAGACAATCAC	GCAAAGATTAGCCTCTGCTGGTC	Ubiquitin-conjugating enzyme related	Transporting ubiquitin
P6[Z]	*SeTIP41*	CCCAGCAAGAGGCTTACGAAG	CCTTCCATCTTTTGCGCCTATCAC	TAP42 interacting protein of 41 kDa related	Participation in the Target-of-Rapamycin (TOR) pathway, which modifies cell growth in response to nutrient status and environmental conditions
P7[Z]	*SeCAC*	GAGCTTAGTCTACCCAGC ATCAC	GCCTGGTATCCACTCTTC TCAATG	Clathrin adaptor complex related	Connection of cargo proteins and lipids to clathrin at vesicle budding sites, as well as binding of accessory proteins that regulate coat assembly and disassembly
P8[X]	*SeNHX1*	GTCCATCACCGCTTTACTAATTGG	GTAATGAAGTTGCGGAAGAATTGC	Specific Tonoplast Na^+/H^+ antiporter	Compartmentalization of Na^+ into vacuoles to alleviate Na^+ toxicity in cytoplasm
P9[X]	*SeVP1*	GAGGTGTTTTCTGCCCTTATGTC	ACAGCCTTGTCATCTAACCGAGT	First vacuolar H^+-PPase	Hydrolysis of cytosolic PP_i to maintain levels and translocation of protons into vacuoles to maintain the acidity of the vacuolar lumen
P10[X]	*SeVP2*	CTTTGTACTCATTTCTGTTGTCATTTTG	ATTGTGCTAGTTAATCTAATGTGGTTGAT	Second vacuolar H^+-PPase	
P11[X]	*SeVHA-A*	CTGGTTCGGATGGTCAAAAGATTAC	CGAGATTGCGGAAAGAAGCACTCA	Catalytic subunit A of the vacuolar H^+-ATPase related	Growth and development of plant cells; maintain acidity of vacuolar lumen
P12[Y]	*SeHKT*	GACATTCGTACCAATCCGCTATG	GCATGATAGAGATGGCCAAGTAAG	Hkt1-like gene	Involved in Na^+ homeostasis under salt stress
P13[Y]	*SeSOS1*	GATGCTCAGCTGCATTGCTTTAG	AGAACATAGCGGACTTCTGAGTTG	Salt overly sensitive 1; Plasma membrane Na^+/H^+ antiporter related	Imparting salt stress tolerance to plants
P14[Y]	*SePerox*	ATGTCCTGGAGTTGTCTCTTGTG	ACCATCTCTTCTTCCGGTTTCC	Peroxidase enzyme	Protecting against salt damage caused by ROS using antioxidant peroxidase
P15[Y]	*SeAAP*	GCTTCATTCTTGTGCTCCTTCC	GCTATTGGTGTGGGTTTGAGTG	Amino acid permease	Synthesis, transportation and accumulation of low-molecular weight organic compounds to improve cellular turgor pressure and cell expansion in the shoots under salt-free conditions
P16[Y]	*SeVinS*	GCTTGGCTAAGGGAAGATTTAAGG	GAACCACCCAAATCCGGTCAC	Vinorine synthase; Synthesis of monoterpenoid indole alkaloid	
P17[Y]	*SeOsmP*	CCCTGTTCGAGTACACTCTGG	CAGCCGTGCAGCTAATCTTAC	Osmotin protein; Low molecular weight hydrophilic protein related	
P18[Y]	*SeProT*	CGATGCCAAAGAGTTCAAGCTC	GACTTGAGGTTGGTTGGGTAATTC	Proline transporter; Osmoregulation compound transporter	
