# Supplementary material for: Molecular analysis of the reactions in Salicornia europaea to varying NaCl concentrations at various stages of development to better exploit its potential as a new crop plant
Source: Front Plant Sci. 2024 Sep 3;15:1454541. doi: 10.3389/fpls.2024.1454541 (PMC11405239; doi:10.3389/fpls.2024.1454541)
Supplement: Supplementary file 1 [file DataSheet1.zip › Supplementary Table 2.pdf]

Supplementary Table 2. Summary of main results of gene expression analysis and determination of total phenolic compounds [µg GAE/ mg FW] and shoot fresh weight [g] in *S. europaea* (74 plants; N.A. for one plant of 30 g/L NaCl treatment in fourth harvest group). Yellow = non-molecular data; red = gene group of transporters that are directly or indirectly involved in Na^{+} transport at the cell membrane or tonoplast; green = group of genes that are directly or indirectly related to oxidative stress; blue = gene group that is related to osmoprotective compounds.

**Analysis of**	**Shoots**	**Roots**
Fresh weight	Increased with development especially in reduced heat stress conditions; Yield according to NaCl concentration [g/L]: 15 > 22.5 > 30 > 7.5 > 0	N.A.
Total phenolic compounds	Peaks at 0 g/L NaCl especially during later development; decreasing with salinity; decreased in mid development	Clearly increasing with development; not influenced by salinity
SeNHX1	Peaks at 0 g/L NaCl; decreasing with salinity; increasing with development	Increased with salinity; slightly increased in early and later development
SeVP1	Increased at 0 g/L NaCl; increased during mid and later development	Decreasing with salinity; slightly increased during mid development
SeVP2	Increased at 0 g/L NaCl; increased during mid development	Decreasing with salinity; clearly increased during mid development
SeVHA-A	Peaks at 0 g/L NaCl; slightly increased during mid and later development	Increased in early development; not influenced by salinity
SeHKT-S	Peaks at 0 g/L NaCl; slightly increased at optimal salinity and during mid and later development	Decreasing with salinity and development
SeSOS1	Peaks at 0 g/L NaCl in mid and later development; lower at near-optimal levels (7.5 and 15 g/L NaCl) compared to extremes	Decreased in later development
SePerox	Peaks at 0 g/L NaCl in mid and later development	Decreasing with salinity
SeAAP	Decreasing in (low-) optimal salinity; Increased during early and late development	Peaks at 0 g/L NaCl; decreasing with development
SeVinS	Peaks at 0 g/L NaCl especially in late development; decreasing with salinity	Decreasing with salinity; decreased in later development
SeOsmP	Peaks at 0 g/L and again but less at 15 g/L NaCl; increasing with development	Peaks at 0 g/L and again but less at 15 g/L NaCl; increasing with development
SeProT	Increased in later development mostly in non-optimal salinity	Decreased in early development
