# Supplementary material for: Molecular analysis of the reactions in Salicornia europaea to varying NaCl concentrations at various stages of development to better exploit its potential as a new crop plant
Source: Front Plant Sci. 2024 Sep 3;15:1454541. doi: 10.3389/fpls.2024.1454541 (PMC11405239; doi:10.3389/fpls.2024.1454541)
Supplement: Supplementary file 1 [file DataSheet1.zip › Supplementary Table 3.pdf]

frontiers

Supplementary Table 3. Summary of ANOVA results of shoot fresh weight, total phenolic compounds and gene expression analysis in *S. europaea* (74 plants in total; N.A. for one plant of 30 g/L NaCl treatment in fourth harvest group). Significant terms (P < 0.05) of the respective variables (Harvest = harvest group; NaCl = NaCl treatment [g/L] and Interaction = Interaction term of both variables) shown in green, non-significant terms in red, missing data is grayed out.

**Analysis**	**ANOVA of shoot data**			**ANOVA of root data**		
	Harvest	NaCl	Interaction	Harvest	NaCl	Interaction
Fresh weight						
Total phenols						
SeNHX1						
SeVP1						
SeVP2						
SeVHA-A						
SeHKT						
SeSOS1						
SePerox						
SeAAP						
SeVinS						
SeOsmP						
SeProT						
