# Supplementary material for: Molecular analysis of the reactions in Salicornia europaea to varying NaCl concentrations at various stages of development to better exploit its potential as a new crop plant
Source: Front Plant Sci. 2024 Sep 3;15:1454541. doi: 10.3389/fpls.2024.1454541 (PMC11405239; doi:10.3389/fpls.2024.1454541)
Supplement: Supplementary file 1 [file DataSheet1.zip › Supplementary Table 4.pdf]

Supplementary Table 4. Summary of mean total phenolic compounds and mean relative gene expression levels of *SeNHX1*, *SeVP1*, *SeVP2*, *SeVHA-A*, *SeHKT*, *SeSOS1*, *SePerox*, *SeAAP*, *SeVinS*, *SeOsmP* and *SeProT* in NaCl treatments [0-30 g/L] averaged over the harvests 35, 50, 65, 80 and 95 d separately for shoots and roots of *S. europaea*. Expression was normalized using mean of *SeUBC*, *SeActin* and *SeDNAJ* reference gene expression and RNA concentrations. Data represents mean ± SE over 15 replicates (74 plants in total; N.A. for one plant of 30 g/L NaCl treatment in fourth harvest group). Similar letters denote no significant difference ($P < 0.05$).

			Mean relative gene expression										
NaCl [g/L]	Organ	Phenolic compounds [µg GAE/mg FW]	*SeNHX1*	*SeVP1*	*SeVP2*	*SeVHA-A*	*SeHKT*	*SeSOS1*	*SePerox*	*SeAAP*	*SeVinS*	*SeOsmP*	*SeProT*
0	Shoot	$0.81^{b} \pm 0.13$	$2.20^{b} \pm 0.48$	$2.92^{b} \pm 0.50$	$1.66^{a} \pm 0.49$	$1.67^{a} \pm 0.48$	$3.17^{b} \pm 0.61$	$1.24^{b} \pm 0.22$	$2.91^{b} \pm 0.81$	$0.71^{a} \pm 0.21$	$4.81^{b} \pm 0.72$	$1.51^{b} \pm 0.50$	$0.93^{a} \pm 0.17$
7.5	Shoot	$0.52^{a} \pm 0.05$	$1.26^{a} \pm 0.15$	$1.52^{a} \pm 0.13$	$1.02^{a} \pm 0.12$	$0.79^{a} \pm 0.07$	$0.93^{a} \pm 0.10$	$0.49^{a} \pm 0.06$	$0.51^{a} \pm 0.08$	$0.38^{a} \pm 0.07$	$1.34^{a} \pm 0.12$	$0.29^{a} \pm 0.07$	$0.49^{a} \pm 0.07$
15	Shoot	$0.44^{a} \pm 0.04$	$1.05^{a} \pm 0.17$	$1.54^{a} \pm 0.17$	$0.87^{a} \pm 0.12$	$0.80^{a} \pm 0.09$	$1.20^{a} \pm 0.25$	$0.52^{a} \pm 0.06$	$0.47^{a} \pm 0.07$	$0.38^{a} \pm 0.07$	$1.24^{a} \pm 0.24$	$0.86^{ab} \pm 0.27$	$0.58^{a} \pm 0.09$
22.5	Shoot	$0.42^{a} \pm 0.06$	$1.02^{a} \pm 0.14$	$1.77^{a} \pm 0.16$	$1.10^{a} \pm 0.14$	$1.00^{a} \pm 0.11$	$1.32^{a} \pm 0.19$	$0.78^{a} \pm 0.11$	$0.48^{a} \pm 0.10$	$0.59^{a} \pm 0.10$	$1.19^{a} \pm 0.16$	$0.28^{a} \pm 0.07$	$0.95^{a} \pm 0.19$
30	Shoot	$0.41^{a} \pm 0.03$	$0.95^{a} \pm 0.08$	$1.53^{a} \pm 0.16$	$0.80^{a} \pm 0.09$	$1.00^{a} \pm 0.12$	$0.99^{a} \pm 0.09$	$0.77^{a} \pm 0.11$	$0.50^{a} \pm 0.08$	$0.59^{a} \pm 0.09$	$0.87^{a} \pm 0.09$	$0.57^{a} \pm 0.28$	$0.87^{a} \pm 0.13$
0	Root	$0.64^{a} \pm 0.05$	$0.68^{a} \pm 0.06$	$1.35^{b} \pm 0.11$	$1.34^{ab} \pm 0.17$	$0.83^{a} \pm 0.05$	$2.05^{a} \pm 0.37$	$0.74^{a} \pm 0.05$	$1.01^{b} \pm 0.11$	$0.74^{b} \pm 0.07$	$0.96^{b} \pm 0.11$	$1.39^{c} \pm 0.32$	$0.72^{a} \pm 0.11$
7.5	Root	$0.64^{a} \pm 0.06$	$0.68^{a} \pm 0.04$	$1.23^{ab} \pm 0.12$	$1.60^{b} \pm 0.21$	$0.82^{a} \pm 0.04$	$1.59^{a} \pm 0.28$	$0.67^{a} \pm 0.03$	$0.61^{a} \pm 0.09$	$0.43^{a} \pm 0.07$	$0.77^{ab} \pm 0.08$	$0.34^{ab} \pm 0.11$	$0.84^{a} \pm 0.09$
15	Root	$0.64^{a} \pm 0.05$	$0.73^{a} \pm 0.07$	$1.08^{ab} \pm 0.11$	$1.16^{ab} \pm 0.20$	$0.72^{a} \pm 0.07$	$1.63^{a} \pm 0.31$	$0.63^{a} \pm 0.06$	$0.56^{a} \pm 0.12$	$0.53^{ab} \pm 0.12$	$0.57^{a} \pm 0.08$	$0.90^{bc} \pm 0.32$	$0.69^{a} \pm 0.08$
22.5	Root	$0.59^{a} \pm 0.05$	$0.74^{a} \pm 0.07$	$1.11^{ab} \pm 0.09$	$1.24^{ab} \pm 0.27$	$0.78^{a} \pm 0.08$	$1.62^{a} \pm 0.32$	$0.67^{a} \pm 0.06$	$0.47^{a} \pm 0.10$	$0.53^{ab} \pm 0.12$	$0.59^{a} \pm 0.10$	$0.20^{ab} \pm 0.07$	$0.74^{a} \pm 0.06$
30	Root	$0.66^{a} \pm 0.05$	$0.95^{b} \pm 0.05$	$0.96^{a} \pm 0.06$	$0.92^{a} \pm 0.09$	$0.68^{a} \pm 0.06$	$1.04^{a} \pm 0.11$	$0.70^{a} \pm 0.05$	$0.40^{a} \pm 0.06$	$0.35^{a} \pm 0.06$	$0.61^{a} \pm 0.08$	$0.14^{a} \pm 0.08$	$0.72^{a} \pm 0.08$
